# Foliar nitrogen metabolism of adult Douglas-fir trees is affected by soil water availability and varies little among provenances

**DOI:** 10.1371/journal.pone.0194684

**Published:** 2018-03-22

**Authors:** Baoguo Du, Jürgen Kreuzwieser, Michael Dannenmann, Laura Verena Junker, Anita Kleiber, Moritz Hess, Kirstin Jansen, Monika Eiblmeier, Arthur Gessler, Ulrich Kohnle, Ingo Ensminger, Heinz Rennenberg, Henning Wildhagen

**Affiliations:** 1 College of Life Science and Biotechnology, Mianyang Normal University, Mianyang, China; 2 Chair of Tree Physiology, Institute of Forest Sciences, Albert-Ludwigs-Universität Freiburg, Freiburg, Germany; 3 Karlsruhe Institute of Technology (KIT) Campus Alpin, Institute of Meteorology and Climate Research (IMK), Atmospheric Environmental Research (IMK-IFU), Garmisch-Partenkirchen, Germany; 4 Department of Biology, Graduate Programs in Cell & Systems Biology and Ecology & Evolutionary Biology, University of Toronto, Mississauga, Ontario, Canada; 5 Forest Research Institute Baden-Württemberg (FVA), Freiburg, Germany; 6 Institute of Medical Biometry, Epidemiology and Informatics (IMBEI), University Medical Center Mainz, Mainz, Germany; 7 Institute for Landscape Biogeochemistry, Leibniz Centre for Agricultural Landscape Research (ZALF), Müncheberg, Germany; 8 Institute of Ecology, Leuphana University of Lüneburg, Lüneburg, Germany; 9 Berlin-Brandenburg Institute of Advanced Biodiversity Research (BBIB), Berlin, Germany; 10 Swiss Federal Research Institute WSL, Birmensdorf, Switzerland; 11 King Saud University, Riyadh, Saudi Arabia; Austrian Federal Research Centre for Forests BFW, AUSTRIA

## Abstract

The coniferous forest tree Douglas-fir (*Pseudotsuga menziesii*) is native to the pacific North America, and is increasingly planted in temperate regions worldwide. Nitrogen (N) metabolism is of great importance for growth, resistance and resilience of trees. In the present study, foliar N metabolism of adult trees of three coastal and one interior provenance of Douglas-fir grown at two common gardens in southwestern Germany (Wiesloch, W; Schluchsee, S) were characterized in two subsequent years. Both the native North American habitats of the seed sources and the common garden sites in Germany differ in climate conditions. Total and mineral soil N as well as soil water content were higher in S compared to W. We hypothesized that i) provenances differ constitutively in N pool sizes and composition, ii) N pools are affected by environmental conditions, and iii) that effects of environmental factors on N pools differ among interior and coastal provenances. Soil water content strongly affected the concentrations of total N, soluble protein, total amino acids (TAA), arginine and glutamate. Foliar concentrations of total N, soluble protein, structural N and TAA of trees grown at W were much higher than in trees at S. Provenance effects were small but significant for total N and soluble protein content (interior provenance showed lowest concentrations), as well as arginine, asparagine and glutamate. Our data suggest that needle N status of adult Douglas-fir is independent from soil N availability and that low soil water availability induces a re-allocation of N from structural N to metabolic N pools. Small provenance effects on N pools suggest that local adaptation of Douglas-fir is not dominated by N conditions at the native habitats.

## Introduction

Douglas-fir (*Pseudotsuga menziesii*) is native to America’s Pacific Northwest (USA, Canada), where it grows in more than 20 million ha of natural forests [1]. Reflecting the great natural range of distribution in longitude, latitude and altitude (from California to British Columbia, and from sea level up to an altitude of >2,000 m), a variety of different ecotypes evolved. These ecotypes are locally adapted, i.e. phenotypes of adaptive traits vary in a way providing highest fitness in the native environments [2–4].

Based on knowledge on intraspecific variation in stress responses and growth, suitable Douglas-fir provenances have been increasingly planted outside North America, e.g. in Central Europe [5,6], New Zealand, Australia, and South America [6]. Available studies on intraspecific variation of Douglas-fir focused on growth [7–11], phenology [12], and photosynthetic gas exchange [13,14], but little is known about natural genetic variation in nitrogen (N) metabolism in field-grown Douglas-fir. N is an important factor for the growth and vitality of trees [15–19]. Differences in climatic, edaphic and biotic factors across large natural ranges of conifer tree species presumably translate into variation in N cycling and, thus, N availability at the native habitats [20–22]. In turn, variation in N availability may cause local adaptation at the level of N uptake [22,23] or metabolism, which is reflected in constitutive variation in N pools among provenances as reported for Norway spruce [20] and Scots pine [24,25]. In a provenance trial with Scots pine, highest needle arginine concentrations were found in the northernmost provenances [25], possibly as a consequence of nutrient imbalances or as an adaptation enabling a C-efficient assimilation of plant available N not required for growth [26–28]. Higher needle total N contents of Norway spruce [20] and Scots pine [24] at higher altitudes or longitudes, or at sites with a cooler climate were concluded to be adaptive [20,24], since they often go along with larger quantities of Rubisco [29,30]. This, in turn, might enable higher net C assimilation rates, which might compensate for environmental limitations for carbon gain.

Adaptation to local environments is very common in trees, and adaptive phenotypic variation is often reflected in genetic differentiation among populations [31]. If genetic differentiation in loci underlying phenotypic variation is constrained by high gene flow, or in temporally highly variable environments, phenotypic plasticity in adaptive traits can confer fitness advantages [32]. Phenotypic plasticity, i.e. the ability of a genotype to express different phenotypes in different environments, may -or may- not vary among genotypes (or populations) [33]. Benomar et al. [34] did not observe variation in plasticity of adaptive traits such as height growth, photosynthetic CO_2_ assimilation and leaf N concentration among seed sources of *Picea glauca*. In Douglas-fir, plasticity in height growth varied among provenances (i.e. the provenance-environment interaction effect was significant) [11], but we lack knowledge on variation in plasticity of N metabolism in Douglas-fir.

Besides variation in N pools as a consequence of genotypic differentiation or genotypic variation in plasticity, N metabolism of trees is also affected by acclimatization to variation in environmental conditions [35,36]. Water shortage and elevated temperature exert strong effects on tree N metabolism [37,38]. Under drought and / or heat, foliar total amino acid contents, particularly amino acids from the glutamate family (e.g. glutamate, arginine), increased in deciduous [39,40] and conifer species [35,41–43]. Accumulation of arginine in needles was regarded as a general response of conifers to stresses causing a reduction in growth [26]. For *Pinus halepensis*, a decreased content of soluble proteins under drought was reported [44], a response reflecting either a shift in N metabolism towards accumulation of osmolytic amino compounds, and / or a reduction of maintenance and synthesis of proteins [45].

In the present study, we characterized the N metabolism of 50-year-old Douglas-fir trees of four provenances grown at two field sites of a common garden experiment located in Germany [46]. The field sites were established in 1961 and differ in annual temperature and precipitation. Trees of one interior and three coastal provenances were grown from seeds collected at North American native habitats differing in precipitation and temperature conditions. The assumption of local adaptation to conditions at the native habitat is supported by significant variation in photosynthetic gas exchange [13] and growth [11] among the provenances studied here. Genetic differentiation of the studied provenances was reported earlier based on both microsatellite [11] and SNP marker analysis [47]. This experimental setting and repeated sampling over the course of two years allowed to study the extent of phenotypic variation in N pools and to attribute this variation to i) differences in environmental conditions among sites and within sites over time, ii) provenance effects reflecting genetic differentiation, or iii) provenance-environment interaction effects (i.e. variation in phenotypic plasticity among provenances). We hypothesized that provenances show constitutive differences in sizes and / or composition of N pools; specifically, we expected that as a consequence of a cooler and dryer climate at their native habitat, the interior provenance shows a higher content of metabolic N including total soluble proteins and highly abundant N-rich amino acids such as arginine and asparagine. We further hypothesized that sizes and composition of N pools are affected by variation in environmental conditions at the common garden sites; in particular, we expected a reduction of structural N and total soluble proteins, and an increase of total amino acids and arginine under reduced soil water availability. Lastly, we hypothesized that effects of environmental factors on N pools differ among provenances; in this context, we expected that the interior provenance shows a smaller extent of re-allocation of structural N towards amino acids due to constitutively higher levels of metabolic N. To analyze the trees’ N metabolism, we determined the total N content as well as the partitioning of N into amino acids, soluble protein and structural N in needles of Douglas-fir provenances by N metabolite profiling.

## Materials and methods

### Plant material and experimental design

Douglas-fir (*Pseudotsuga menziesii* (Mirbel) Franco) trees of three coastal provenances (var. *menziesii*; ‘WA Darrington 3 Conrad Creek’ (CR), ‘BC Cameron Lake’ (LA), ‘OR Santiam River’ (RI)) and one provenance of the interior variety (var. *glauca*; ‘BC Salmon Arm’ (AR)) were studied. For geographical maps of the origin of these four provenances and the two field sites see Müller et al. [47]. Selection criteria for the provenances were 1) presence on both study sites and 2) high variation in climatic conditions of their natural sites (Table 1) [46]. Long-term averages (period 1961–1990) of yearly climatic moisture deficit (CMD) reveal that the interior provenance AR must acquire more water from other sources than rain (i.e. soil moisture) to avoid the impact of drought stress compared to coastal provenances [48]. CMD data for sites of origin were retrieved from ClimateNA version 5.50 for CR and RI, and from ClimateBC version 5.50 for AR and LA [49].

**Table 1:** Climatic and geographical features of the origin sites of the four provenances.

Prove-nance	Geographical area	Elevation (m a.s.l.)	Precipitation (mm)		Temperature (°C)		Climatic moisture deficit^3^ (mm)
			^1^	^2^	^1^	^2^	
Salmon **Ar**m (AR)	Southern inland, Canada (119°13′W, 50°39′N)	580	500	205	7.8	15	389±86
Conrad **Cr**eek (CR)	North Cascades, US (121°30′W, 48°15′N)	280	2300	518	9.5	14	213±70
Cameron **La**ke (LA)	Vancouver Island, Canada (124°40′W, 49°15′N)	210	1475	320	10.0	15	225±60
Santiam **Ri**ver (RI)	West Cascades, US (121°58′W, 44°40′N)	800	1780	410	9.5	14.5	370±86

^1^Mean annual,

^2^Growing season, Data from: Kenk and Thren [46].

^3^For calculation see [48].

Data represent mean ± standard deviation for the yearly CMD for the period 1961–1990.

The trees studied were grown at two field sites in Germany, i.e. “Wiesloch” (“W”) and “Schluchsee” (“S”), that had been established in 1961 as part of an international provenance trial [46]. Seeds collected for this trial originated from open-pollinated trees in naturally regenerated stands in British Columbia (Canada), Washington, and Oregon (USA) [46] as previously described by Neophytou et al. [11]. At each field site, each of the selected provenances had been planted at a density of 3,300 trees ha^-1^ on two to four plots (each 0.1 ha). The plots had been arranged irregularly, so that identical provenances did not adjoin [11,50]. A height-driven thinning regime has been applied (see Kohnle et al. [50] for details). At the end of the growing season 2011, trees reached heights between 26.6±0.3 m (AR at S) and 33.7±0.3 m (CR at W) [11]. Annual precipitation, precipitation during the vegetation period, mean annual temperature and CMD differ between both sites (Table 2). Long-term averages (period 1961–1990) of CMD are within the range of the native habitats (site W) (Table 1) or lower (site S). CMD data for the field sites in Germany were retrieved with the ClimateEU version 4.63 software package (based on the methodology described by [51]), available at http://tinyurl.com/ClimateEU.

**Table 2:** Climatic and geographical features of the two selected field sites in Germany.

Sites	Geographical area	Soil type and pH	Elevation (m a.s.l.)	Precipitation (mm)		Temperature (°C)		Climatic moisture deficit^3^ (mm)
				^1^	^2^	^1^	^2^	
Wiesloch (W)	Upper Rhine lowland	Sandy clay, upper shell limestone with aeolic loess deposits, pH ~ 6.6 (data from [52])	105	660	336	9.9	17.0	226±79
Schluchsee (S)	Black forest	Well-drained iron-humus-podzol, pH ~3.3 (data from [53])	1050	1345	590	6.1	12.7	43±39

^1^Mean annual,

^2^Growing season, Data from: Kenk and Thren [46];

^3^Data represent mean ± standard deviation for the yearly CMD for the period 1961–1990.

Weather data in one-hour-resolution for the growing seasons of 2010 and 2011 were available from a private meteorological station at Schluchsee (N47°49’16”, E8°11’08”) and from Germany’s National Meteorological Service (Deutscher Wetterdienst) station Kirrlach-Waghäusel (N49°15’0”, E8°32’24”) which is located in close proximity to the site Wiesloch. Soil water availability was modelled in daily resolution using the forest hydrological water budget model WBS3 that estimates daily total available soil water (TAW) using temperature, precipitation, latitude, soil type, plant cover, slope, and slope aspect [54].

### Soil sampling and chemical analyses

Soil samples were collected in two layers, 0–10 cm and 10–20 cm, using a hand auger at the studied plots in W and S in August 2013. The litter was manually removed before sampling. Soil samples were stored in plastic bags at 4°C before passed through a sieve (mesh width 2 mm) for further analyses. Half of the samples were dried at 60°C in a drying oven until reaching constant weight. Dried soil was powdered in a ball mill (MM400, RETSCH GmbH, Haan, Germany) and used for analysis of total N and carbon (C) concentrations as well as for analysis of δ^15^N. The samples were weighed and placed in tin capsules (5 × 9 mm; IVA Analysentechnik e.K., Meerbusch, Germany). Analyses were conducted using a Costech Elemental Analyzer (Costech International S.p.A., Milano, Italy) equipped with a zero-blank auto-sampler and coupled via a ConFloIII to a Thermo Finnigan Delta V Plus isotope ratio mass spectrometer (Thermo Scientific, Waltham, MA, USA) as described in detail by Liu et al. [55].

Ammonium and nitrate were extracted using field-fresh moist soil according to the method described by Schlotter et al. [56]. Fresh soil samples were extracted with distilled water (1:2, w/w) and shaken at 4°C for 24 hours. Extracted solutions were then centrifuged and the supernatants were filtered. Nitrate contents were analyzed with an ion chromatograph (DX 120, Dionex, Idstein, Germany) connected to an autosampler (AS 3500, Thermo Separation Products, Piscataway, NJ, USA). Concentrations were calculated using external nitrate standards. Ammonium was analyzed photometrically (UV-DU 650 spectrophotometer, Beckman Coulter Inc., Fullerton, CA, USA) at 650 nm as based on the color reaction between ammonium and a weakly alkaline mixture of sodium (Na) salicylate and Na hypochlorite as a chlorine source in the presence of Na nitroprusside [57]. Oven-dried ammonium sulfate was used as standard for quantification.

### Sampling and processing of needles

Needles of five to six individual trees per provenance were sampled. All trees sampled were part of the original establishment of the site in 1961. Previous year needles were sampled in late spring (W: May 12^th^ 2010, May 12^th^ 2011; S: May 27^th^ 2010, May 26^th^ 2011) and in summer (W: July 14^th^ 2010, July 14^th^ 2011; S: July 28^th^ 2010, July 28^th^ 2011) at each site. Campaigns at W were carried out prior to campaigns at S to account for the later start of the growing season at S [13]. Needles were sampled from the south-facing upper canopy of the trees using hydraulic platforms, pre-installed ropes or a shotgun in the early afternoon between 1 pm and 3 pm. Needles were rapidly excised from the twigs, immediately shock-frozen in liquid nitrogen, homogenized to a fine powder under liquid nitrogen and subsequently stored at -80 °C until analysis. For determination of needle water content, aliquots of 100 mg frozen powder were transferred to 1.5 ml reaction tubes and were dried at 60°C until reaching weight constancy. Needle water content was calculated as the difference between fresh and dry weight.

### Determination of N pools and compounds in needles

Total N, total soluble protein (TSP), amino compounds and ammonium in needles were determined as previously described [58]. Total N concentration in oven-dried needle powder was determined after Kjeldahl digestion and analyzed on a AT200 spectrophotometer (Beckman Coulter / Olympus, USA) at 630 nm. TSP content was analyzed as previously described by Dannenmann et al. [59] with some adaptations for Douglas-fir needles [58]. For extraction, 35 mg frozen needle powder was added to a mixture of 1.5 ml extraction buffer (50 mM Tris–HCl, 1 mM ethylenediaminetetraacetic acid, 15% glycerol (v/v), 1 mM phenylmethylsulfonyl fluoride, 5 mM dithiothreitol, 0.1% Triton X-100; pH 8.0) and 150 mg polyvinylpyrrolidone. After 30 min incubation with shaking at 4°C followed by centrifugation at 14,000 g for 15 min at 4°C, 500 μl aliquots of the supernatant were transferred to new tubes. The pellet was extracted again with 0.5 ml extraction buffer, and the supernatants were combined thereafter. To precipitate dissolved proteins, 10% (w/v) trichloroacetic acid was added to the mixed supernatant in the same volume as the combined supernatants. The mixture was incubated for 10 min at 4°C. After centrifugation, the pellets were dissolved in 0.5 ml 1 M KOH. Five μl aliquots of the protein extract were mixed with 200 μl Bradford reagent (Amresco Inc., Solon, Ohio, USA). Absorbance at 595 nm was measured by a Sunrise Microplate Reader (Tecan Austria GmbH, Grödig, Austria) and calibrated with bovine serum albumin (BSA; Sigma-Aldrich Chemie GmbH, Germany) standards.

Amino acids and ammonium were analyzed using an ultra-performance-liquid-chromatography (UPLC) system (Waters Corp., Milford, MA) as described by Winter et al. [60] and Luo et al. [61]. Approximately 50 mg ground plant material was extracted in 1 ml chloroform/methanol (3/7, v/v) and 0.2 ml extraction buffer (20 mM Hepes, 5 mM EGTA, 10 mM NaF, pH 7.0) on ice. After 30 min incubation, 600 μl *dd*H_2_O were added to the samples, mixed, and centrifuged for 10 min, 14,000 g at 4°C. Aliquots of supernatants were transferred to new tubes, and 600 μl *dd*H_2_O were again added to the first extraction tube followed by 10 min centrifugation. Supernatants from the combined aqueous phases were freeze-dried (Alpha 2–4, Christ, Osterode, Germany) for 3 d. Amino acids and ammonium were separated on an AccQ-Tag Ultra column (2.1×100 mm; Waters) using a gradient of AccQ-Tag Ultra eluents A and B (Waters) at a flow rate of 0.7 ml min^-1^. The absorbance at 260 nm was detected and quantified using amino acid and ammonium standards.

### Structural N and total amino acids calculation in needles

Structural N content in needles was calculated by subtracting the N fractions of soluble proteins, amino acids and ammonium from total N. According to Junker et al. [13], N amounts of chlorophyll in Douglas-fir needles were as low as 1.8% and, therefore, were neglected in the calculation. Total amino acids (TAA) contents were calculated as the sum of the concentrations of individual amino acids determined as described above.

### Statistical analysis of data from soil and needle samples

For an initial exploratory analysis of needle N compounds, the publicly available platform “MetaboAnalyst 3.0” (http://www.metaboanalyst.ca/MetaboAnalyst/) [62] was used for Partial Least Squares Discriminant Analysis (PLS_DA). Raw data were cube root transformed and auto-scaled (mean-centered and divided by the standard deviation) because of different ranges and variances of the variables. Missing values were processed with an inbuilt function of MetaboAnalyst which excluded features with >50% missing values and replaced the remaining missing values by the minimum value of the same feature.

For total N, TSP, structural N, TAA, and the three most abundant amino acids arginine, glutamate, and asparagine potential effects of provenance and environmental factors were analysed by linear modelling using the statistical computing environment R v3.3.1 [63]. Based on graphical inspection of the distribution of the data, modelling was done with untransformed data of total N, structural N and glutamate, log2-transformed data of TAA, arginine and asparagine, and square-root transformed data of total soluble protein. As suggested by Zuur et al. [64], we started modelling with a possibly beyond-optimal model including ‘provenance’, ‘site’, ‘TAW’, ‘year’ and ‘season’ as fixed main effects and the interaction effects ‘provenance:site’, ‘provenance:TAW’, ‘site:year’. We considered ‘TAW’ to represent variation in soil water availability within and between sites. To represent variation between sites not directly related to water availability, but to e.g. nutrient concentrations or long-term acclimatization to site-specific environments, we included ‘site’ as a predictor in addition to ‘TAW’. We included ‘season’ (encoded as daylength of sampling day centered to summer solstice as described in Hess et al. [65]) as a predictor to account for effects brought about e.g. by photoperiod, which are independent from ‘TAW’ and ‘site’. ‘Year’ was included as variable integrating variation in environmental conditions over the year of study.

With the above fixed effects model structure, we compared models with a random intercept for tree (function ‘lme’, R package ‘nlme’; [66]) against models without random intercept (function ‘gls’) by BIC and LRT-test. For all dependent variables, models without random effect provided a better fit to the data (dBIC >2). Consequently, the selection of the fixed effects structure was performed on the models without random effect (function ‘gls’) by iteratively dropping non-significant fixed effects (P > 0.01) with highest P-values as computed with the function ‘anova’. This procedure was iterated until all explanatory variables were significant (P < 0.01).

To calculate variance proportions of each significant explanatory variable, the final models were re-fitted using the function ‘lm’, and the proportion of explained variance of a given predictor was calculated by dividing the sum of squares of that predictor by the total sum of squares. Normal distribution and homogeneity of variance of the residuals of the final models were confirmed graphically.

To avoid confounding effects of ‘site’, ‘season’ or ‘year’ on effects of ‘TAW’, we analysed their degree of collinearity. Collinearity of ‘year’ and ‘season’ with ‘TAW’ was small (r^2^ = 0.1/r = 0.31 and r^2^ = 0.16/r = 0.4, respectively), while collinearity of ‘TAW’ and ‘site’ was higher (r^2^ = 0.54/r = 0.73). This prompted us to calculate variance inflation factors (VIFs; calculated with function ‘VIF’, package ‘fmsb’ [67]), which are a commonly used indicator for inaccurate modelling results as a consequence of collinearity (VIFs > 10 indicate distorted model estimation; [68]). VIFs for the starting models and final models ranged from 1.2 to 4.0 (see Results section), indicating reliable model estimation. To further dissect effects of ‘TAW’ and effects of ‘site’ independent from water availability, we used sequential regression. This method is recommended to compute predictors that are completely independent [68]. To achieve this, explanatory variables are regressed against each other [68]. We considered ‘TAW’ as the most direct and obvious indicator for water availability and thus regressed site on ‘TAW’ (‘site’ ~ ‘TAW’) using a generalized linear model (function ‘glm’, binomial family, logit link). The residuals of this regression are orthogonal to ‘TAW’ are thus considered to represent site effects not directly related to water availability. We then modelled (function ‘lm’) N compounds as functions of ‘TAW’ and the residuals (on response scale) of ‘site’ ~ ‘TAW’ (referred to as resid (‘site’)): N compound ~ ‘TAW’ + resid (‘site’). N compounds were transformed as described above for standard multiple regression.

Statistical analysis of differences in soil N and C contents between the two field sites was done by Student’s T test using the software package SigmaPlot 11.0 (Systat Software GmbH, Erkrath, Germany), and raw data were transformed by denary logarithm to match normal distribution if necessary.

## Results

### Soil nitrogen and environmental conditions during sampling campaigns varied considerably between field sites

Contents of total C, total N, nitrate and ammonium in soil from S were considerably higher than from W in both 0–10 cm and 10–20 cm layers, with the exception of ammonium contents in the upper layer (Table 3). The C / N ratios of soil at W were significantly higher than at S in both layers. In the upper layer, soil δ^15^N at S was significantly higher than at W, but no significant difference was found between the two sites in the deeper layers (Table 3).

**Table 3:** Soil nitrogen (N) and carbon (C) status at the two field sites of Wiesloch (W) and Schluchsee (S).

Concentrations (per kg DW)		0–10 cm	10–20 cm
NO_3_^−^ (mg)	W	**7.03±1.07**	**1.74±0.40**
	S	**24.52±5.35**	**19.65±5.42**
NH_4_^+^ (mg)	W	0.23±0.09	**0.06±0.01**
	S	0.30±0.08	**0.22±0.06**
Total N (g)	W	**1.2±0.10**	**0.49±0.04**
	S	**5.25±0.30**	**4.07±0.35**
Total C (g)	W	**22.06±1.06**	**9.29±0.94**
	S	**76.03±4.13**	**59.38±4.62**
C / N ratio	W	**18.43±0.22**	**18.69±0.56**
	S	**14.51±0.17**	**14.68±0.21**
δ^15^N (‰)	W	**-0.05±0.20**	2.13±0.32
	S	**1.13±0.31**	1.71±0.36

Bold values show significant differences (*P* < 0.05) between the two field sites.

The weather conditions during and before the field campaigns varied considerably between the two sites and the years (Table 4). In 2010, yearly CMD of both sites (i.e., 50 mm at S and 207 mm at W) was within the range of the long-term average ± 1 standard deviation, while 2011 (i.e., 97 mm at S and 325 mm at W) was drier at both sites (compare Table 2). In 2010, the conditions were exceptionally warm and dry in July at W, but rather normal (compared to the meteorological reference period) at S and at W in May. Conditions at both sites were warmer and drier in May 2011 than usually. As a consequence of these dry and warm conditions in July 2011 (at W) and May 2011 (at S and W), total available soil water (TAW) was considerably decreased (Table 4). TAW was always higher in S compared to W (Table 4). Data on pre-dawn and midday twig water potential presented in [13] mirror site-related differences in TAW.

**Table 4:** Weather conditions and total soil water availability for each sampling campaign.

Year	Month	Site	P24 (mm)	P168 (mm)	T24 (°C)	TAW (%)
2010	May	W	1.1	8.5	9.3	51.8
2010	July	W	0.1	0.4	22.0	16.4
2010	May	S	1.1	1.2	13.2	82.1
2010	July	S	1.4	4.8	12.9	80.5
2011	May	W	2.7	2.7	18.3	10.0
2011	July	W	5.3	29.5	16.0	19.4
2011	May	S	1.1	27.5	16.0	26.7
2011	July	S	1.8	19.2	13.4	93.7

P: sum of precipitation for a period of 24 hours and 168 hours before the timepoint of harvest (~ 2 p.m. of sampling day). T24: Mean air temperature during the last 24 hours before harvest. TAW: total available soil water on the day of harvest.

### Exploratory analysis suggests effects of site and time-of-the-year on Douglas-fir nitrogen profiles

**Fig 1:**
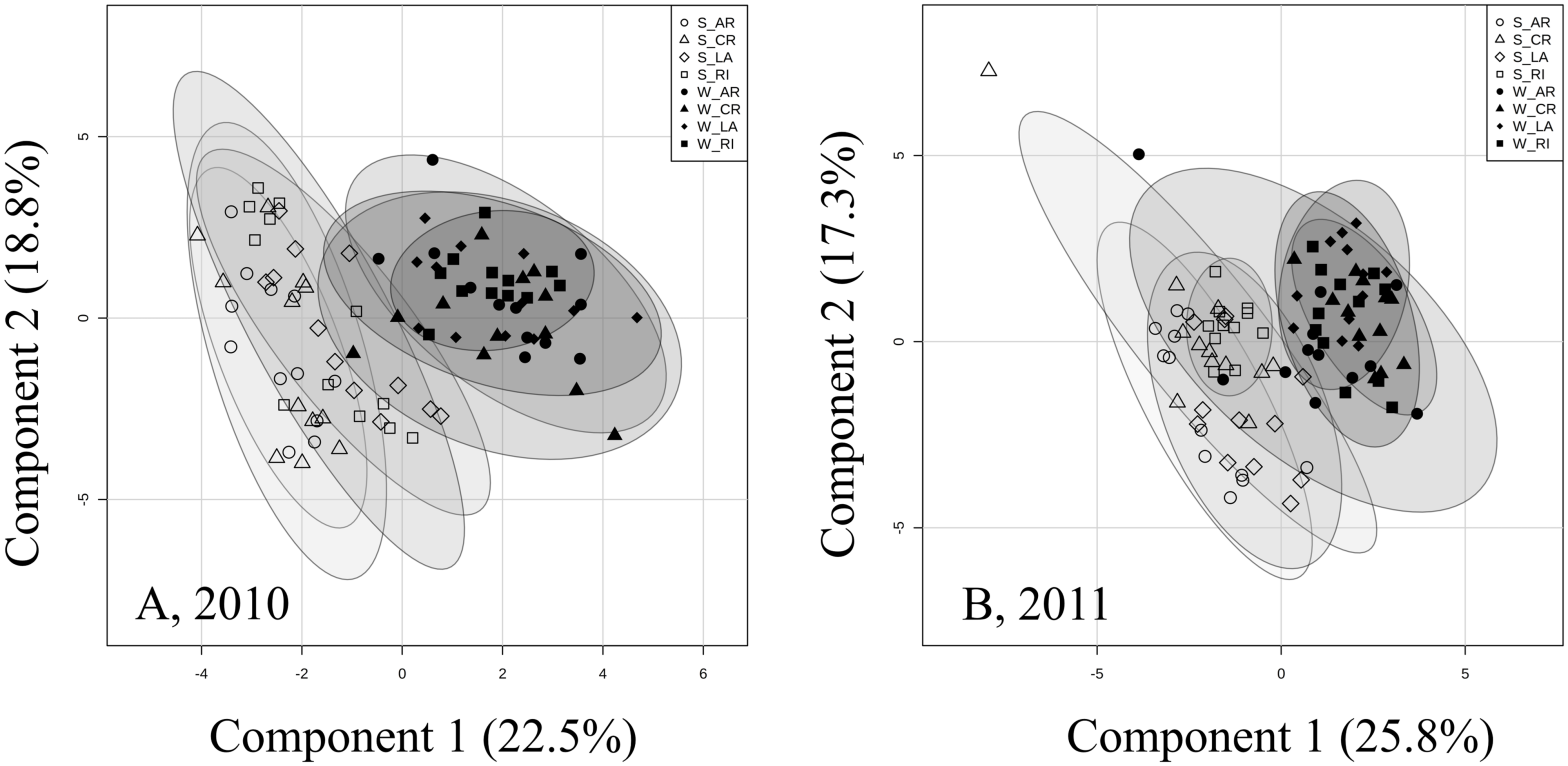
Clustering of N pools and free amino acids in needles of four Douglas-fir provenances. Sampling was done in May and July 2010 (A) and 2011 (B). Figures represent score plots of a partial least squares–discriminant analysis. Douglas-fir provenances (AR, Salmon Arm; CR, Conrad Creek; LA, Cameron Lake; and RI, Santiam River) are represented by different symbols, open and solid symbols indicate individual trees at field sites Schluchsee (S) and Wiesloch (W) irrespective of month of sampling. Semi-transparent shadings indicate 95% confidence regions.

In a first approach to analyze effects of ‘site’, ‘provenance’ and environmental conditions on Douglas-fir N metabolism, we applied a PLS_DA to data of individual amino acids, total and structural N, TSP and TAA. This analysis clearly indicated a clustering of trees according to site in both years (Fig 1A and 1B). As ranked by Variable Importance in Projection (VIP) scores, TAA, soluble protein and arginine were among the five most important compounds determining the site-related clustering in both years (S1A and S1B Fig). Trees at W generally showed higher contents of these components as compared to site S (S1A and S1B Fig). In July 2010, all four provenances at W showed more than 3 times higher proline contents than at S, especially AR with up to 7 times higher contents (S1 Table).

**Fig 2:**
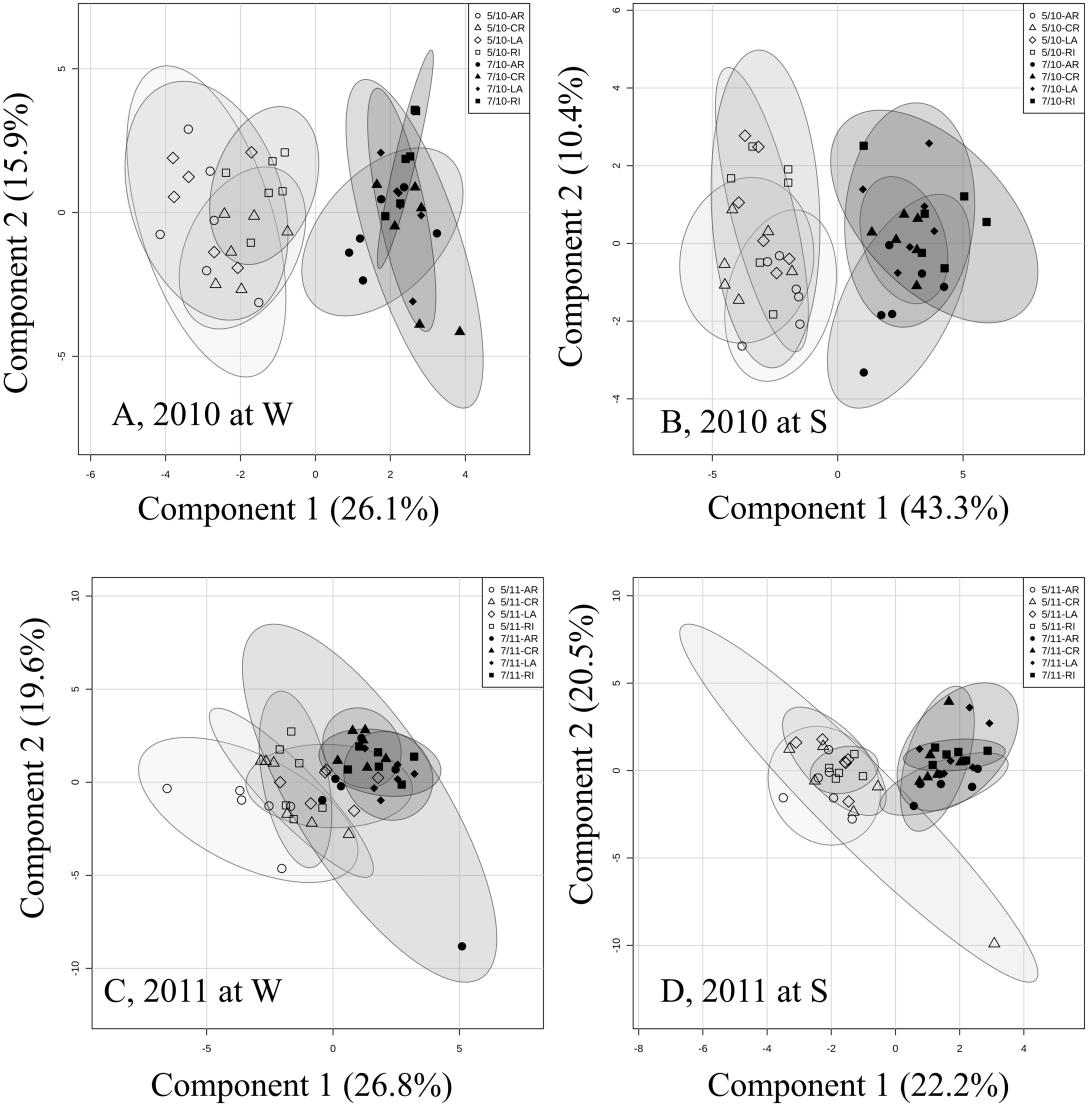
Clustering of N pools and free amino acids in needles computed separately per year. Sampling was done in May and July 2010 (5/10 and 7/10) and 2011 (5/11 and 7/11). Figures represent score plots of a partial least squares–discriminant analysis computed per year and site. A) and C) show plots for Wiesloch (W), B) and D) for Schluchsee (S). Douglas-fir provenances (AR, Salmon Arm; CR, Conrad Creek; LA, Cameron Lake; and RI, Santiam River) are represented by different symbols, open and solid symbols indicate individual trees. Semi-transparent shadings indicate 95% confidence regions.

To investigate any possible clustering according to the month of sampling (May *vs*. July), PLS_DA were computed for data subsets split per site and year (Fig 2A–2D). A clear separation of samples collected in May and July was evident in all four data subsets. Important N compounds determining this clustering differed between years and sites (S2 Fig). Generally, contents of glutamine and proline were higher in May than July in both years irrespective of sites (S2 Fig and S1 Table).

A strong clustering of provenances was not indicated by Figs 1 or 2. PLS_DA computed per site, year and month of sampling supported this finding (S3 and S4 Figs). Clustering of provenances was weak with no consistent patterning (S3 and S4 Figs). At W, there was a tendency that the interior provenance AR differed from the other–coastal–provenances in N profiles. AR showed higher contents of tyrosine and proline than the other three provenances in 2010 and May 2011 at site W (S1 Table).

### Soil water availability and year of study explain most of the variation in needle N pool sizes and concentrations of major amino acids

In order to dissect effects of ‘provenance’, ‘year’, ‘season’, ‘TAW’ (soil water availability integrating environmental factors including temperature, precipitation, and latitude) and ‘site’, we fitted linear models to data on N pool sizes and the most abundant amino acids.

**Fig 3:**
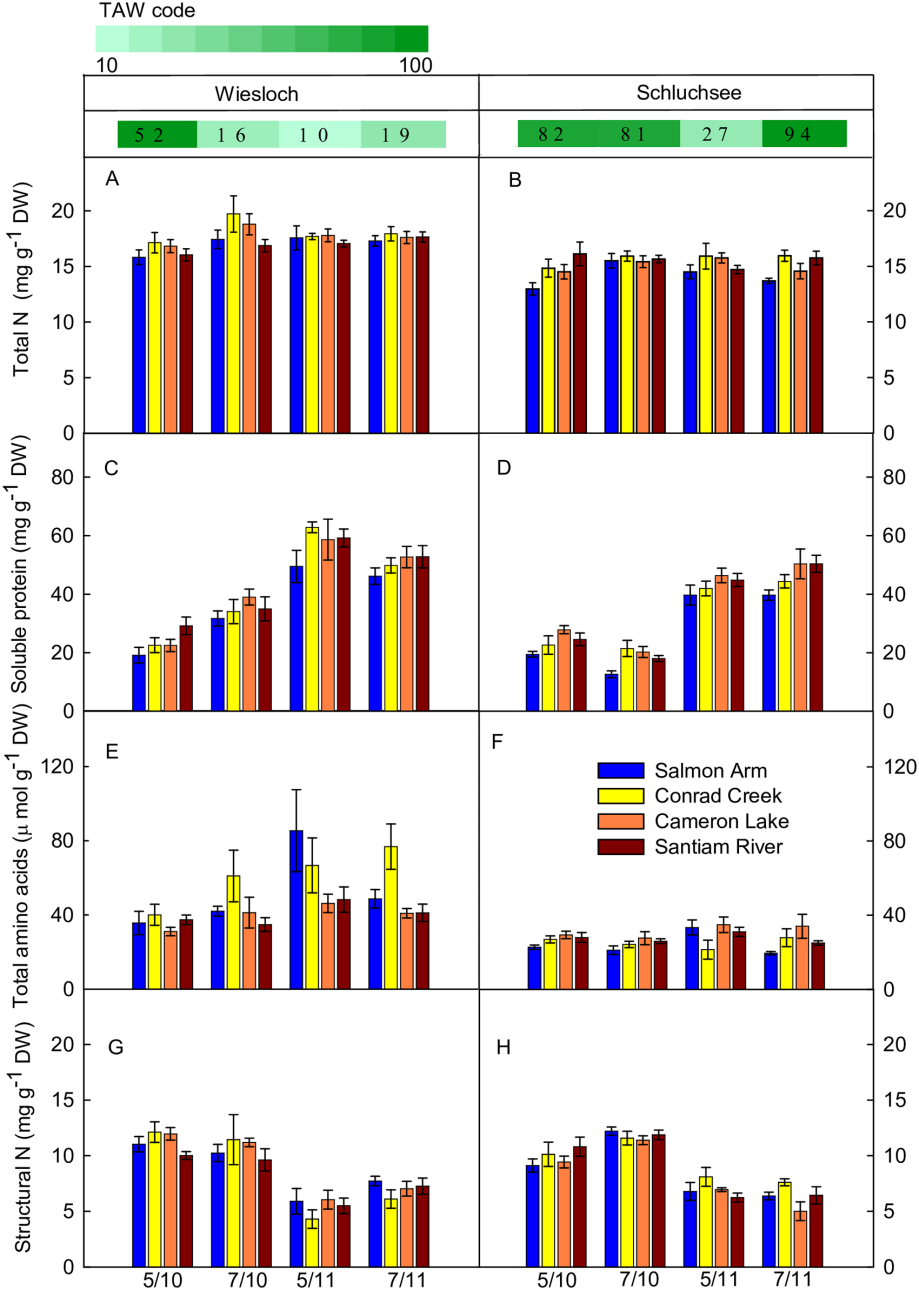
Total N, total soluble protein, total amino acids and structural N contents in needles. Data represent means ± SE of five to six replicates of four Douglas-fir provenances at the two field sites, Wiesloch (left panel) and Schluchsee (right panel), in May and July of 2010 (5/10, 7/10) and 2011 (5/11, 7/11). Stripes above panels indicate modeled total available soil water (TAW in %) at the sampling day.

The total N content of Douglas-fir needles ranged between 13.0 and 19.7 mg g^-1^ DW (Fig 3A and 3B). Linear modelling revealed that there was a small but significant difference in total N content among the provenances, which was caused mainly by higher total N contents in CR and LA as compared to AR (Table 5 and S5A Fig). Most of the variation in total N content was explained by ‘TAW’ (23% of total variation), which is reflected in a significant and provenance-independent trend towards lower total N contents at higher TAW. In addition, total N contents were significantly higher at site W compared to site S, and there was a significant but very small effect of ‘season’, towards higher total N contents in May compared to July.

**Table 5:** Significant explanatory factors for data on N fractions and compounds in needles.

	Model				
Dependent Variable	Explanatory Factor	P-value	Variance proportion	r^2^/r^2^_adj_	VIF
Total N	‘Provenance’	0.003	0.05	0.38/0.36	1.7/1.61
	‘TAW’	< 0.0001	0.23		
	‘Site’	< 0.0001	0.07		
	‘Season’	0.0054	0.03		
Total soluble protein	‘Provenance’	< 0.0001	0.04	0.74/0.73	3.94/3.85
	‘TAW’	< 0.0001	0.25		
	‘Site’	0.001	0.02		
	‘Year’	< 0.0001	0.44		
Structural N	‘TAW’	< 0.0001	0.06	0.54/0.54	2.34/2.18
	‘Site’	< 0.0001	0.05		
	‘Year’	< 0.0001	0.43		
Total amino acids	‘Provenance’^§^	0.0511	0.02	0.49/0.47	2.19/1.94
	‘TAW’	< 0.0001	0.36		
	‘Site’	< 0.0001	0.05		
	‘Provenance:TAW’	0.0005	0.05		
Asparagine	‘Provenance’^§^	0.033	0.04	0.15/0.12	1.22/1.17
	‘TAW’	0.002	0.05		
	‘Provenance:TAW’	0.006	0.06		
Arginine	‘Provenance’	< 0.0001	0.09	0.74/0.72	4.04/3.78
	‘TAW’	< 0.0001	0.41		
	‘Site’	< 0.0001	0.1		
	‘Year’	< 0.0001	0.1		
	‘Provenance:TAW’	0.0008	0.03		
Glutamate	‘Provenance’	0.0064	0.03	0.50/0.49	2.19/2.02
	‘TAW’	< 0.0001	0.27		
	‘Site’^§^	0.5944	0.0		
	‘Year’	< 0.0001	0.16		
	‘Site:Year’	0.0005	0.03		

Needle samples of adult Douglas-fir trees of four provenances growing on two contrasting field sites in south-west Germany were collected in 2010 and 2011. Linear models represent the final models after model selection (see Material and methods for details). Variance proportion: Fraction of total variance explained by the respective factor.

^§^ Main effects with P > 0.01 were kept in the model because they were part of significant (P<0.01) interaction effects. VIF: Variance Inflation Factor (starting model/final model).

Total soluble protein content of needles of the different Douglas-fir provenances showed a relatively large range from 13 to 63 mg g^-1^ DW (Fig 3C and 3D). Like total N, there was a small but significant effect of ‘provenance’ (Table 5 and S5B Fig), due to a lower TSP content in AR as compared to the other provenances. We detected a significantly higher TSP content when TAW was low. There was a small but significant effect towards lower TSP contents in S compared to W. Across all provenances and sites, TSP contents were significantly higher in 2011 compared to 2010, and this effect accounted for 44% of the variation in TSP content.

Structural N content did not differ significantly among the provenances. With increasing TAW, structural N showed a small, significant increase (Table 5 and S5C Fig). Structural N content was significantly higher at W compared to S, and higher in 2010 compared to 2011 on both sites. This variability between the two years under study accounted for 43% of the variation in structural N content.

TAA contents significantly increased from 20 to 85 μmol g^-1^ DW with decreasing TAW (Fig 3E and 3F, Table 5 and S5D Fig). This effect accounted for 36% of the variation in TAA. The effect of ‘TAW’ was to a small extent dependent on provenance with less impacts of TAW on LA compared to the other three provenances. TAA were significantly lower in trees at S as compared to W.

**Fig 4:**
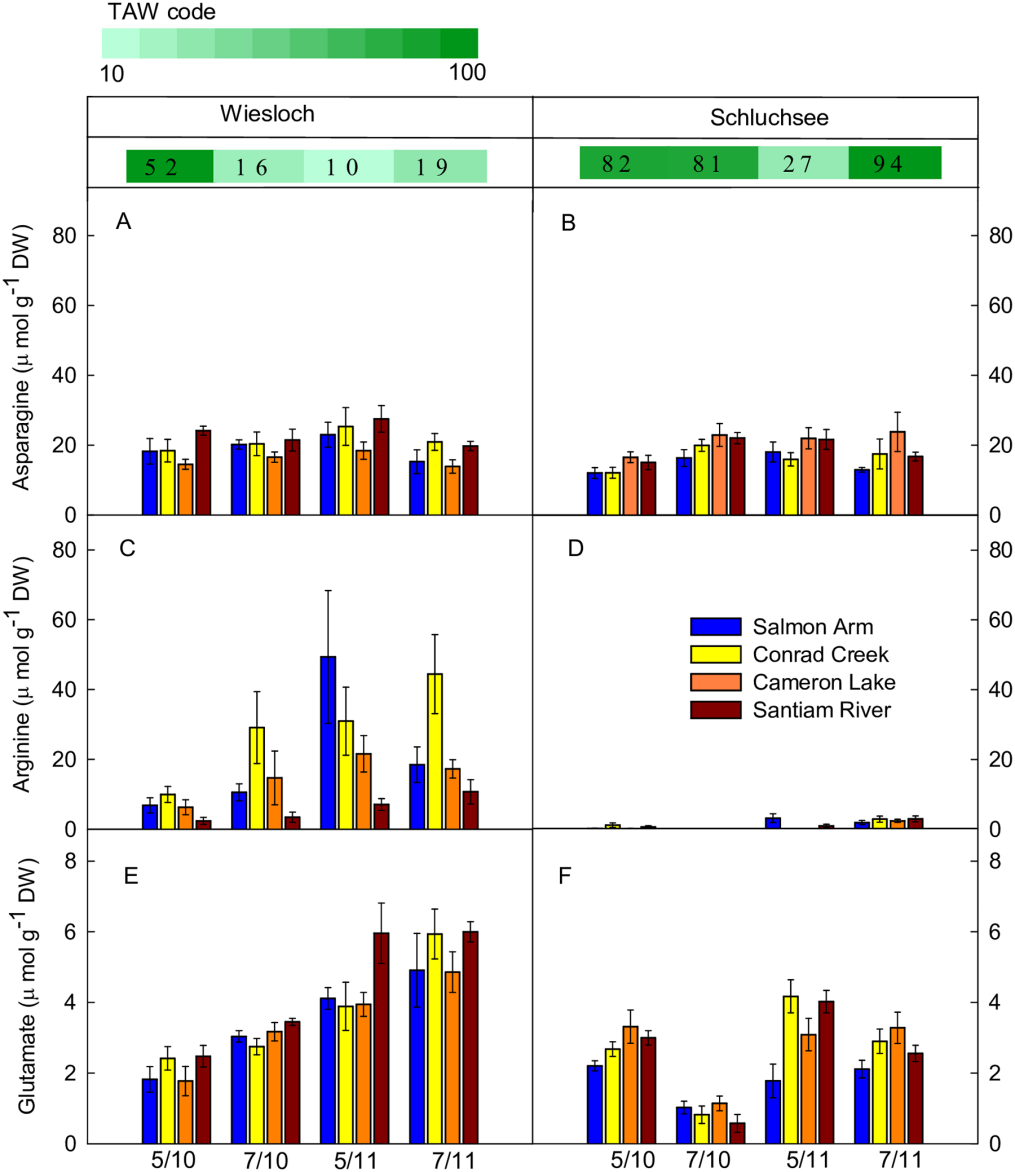
The three most abundant amino acids of four Douglas-fir provenances. Data represent means ± SE of five to six replicates of trees growing at field sites Wiesloch (left panel) and Schluchsee (right panel) in May and July 2010 (5/10, 7/10) and 2011 (5/11, 7/11). Stripes above panels indicate modeled total available soil water (TAW in %) at the sampling day.

The three most abundant amino acids were asparagine, arginine and glutamate (Fig 4) which contributed with up to 93% to the TAA content as observed in provenance CR at W in July 2011. Asparagine contents ranged from 12.05 μmol g^-1^ DW in AR to 27.56 μmol g^-1^ DW in RI (Fig 4A and 4B). Asparagine contents were significantly affected by ‘TAW’ and the interaction (P<0.005) of ‘TAW’ with ‘provenance’ (Table 5 and S5E Fig). While provenances LA and CR were highly fluctuating and did not display a clear pattern to changes in TAW, asparagine contents in AR constantly decreased with increasing TAW, while asparagine contents of RI were unaffected by increasing TAW until it reached 80%.

Arginine contents in trees of the different provenances varied between concentrations below the limit of detection (about 0.13 μmol g^-1^ DW, half of the minimal detected value) and approximately 60 μmol g^-1^ DW (Fig 4C and 4D). This variation among provenances accounted for 9% of the total variance (Table 5 and S5F Fig), and was reflected in significantly lower arginine contents in provenance RI compared to the other provenances. Most of the variation (41%) in arginine contents was explained by the significant trend towards lower arginine concentration with increasing TAW. There was a small but significant interaction effect of ‘TAW’ and ‘provenance’, reflected in a less pronounced decrease of arginine concentrations with increasing TAW in provenance RI. Arginine concentrations were higher in W compared to S: At W, arginine contents ranged from 2.41 to 49.36 μmol g^-1^ DW. By contrast, at field site S, arginine contents showed a maximum of 3.24 μmol g^-1^ DW (CR, May 2011); in July 2010, arginine contents of all provenances were below the limit of detection. Similar to TSP and TAA contents, arginine contents were significantly higher in 2011 as compared to 2010, irrespective of site.

Glutamate contents varied only slightly among provenances, but showed a strong and significant trend towards lower glutamate contents with increasing TAW (Table 5 and S5G Fig). This effect accounted for 27% of the variation in glutamate contents. In W, glutamate contents were significantly higher as compared to S. Moreover, glutamate contents were significantly higher in 2011 as compared to 2010. However, the increase in glutamate contents in 2011 was smaller in S as compared to W, reflected in a small but significant ‘site:year’ interaction effect.

For all modelled N compounds, variance inflation factors were substantially smaller than 10 for both the starting models and the final models, indicating reliable model estimation (Table 5). We confirmed that effects of ‘TAW’ were not confounded by effects of ‘site’ by applying sequential regression (S2 Table). Significance and variance proportions of ‘TAW’ and site effects independent from ‘TAW’ estimated by sequential regressions (S2 Table) were very similar to the results obtained by classical multiple regression (Table 5), corroborating that the effects of ‘TAW’ and ‘site’ reported above were reliably estimated.

## Discussion

### Under low soil water availability, a largely conserved acclimatization of foliar N metabolism involves re-allocation from structural N to metabolic N

Our hypothesis that needle N profiles are affected by variation in environmental conditions at the field sites was supported by both, exploratory and inferential statistical analyses. Linear modelling revealed that total available soil water (TAW) was an important factor (Table 5) underlying variation in N pools between sites indicated by the exploratory analysis (Fig 1 and S1 Fig). The field experiment reported here was complemented by a recently published study of Junker et al. [13] on C metabolism, which revealed a pronounced reduction of photosynthetic CO_2_ assimilation rate, stomatal conductance and midday twig water potential under low TAW. These results show that responses to low TAW were not limited to N metabolism. In agreement with our hypothesis, we observed a trend to higher total amino acids (TAA) and arginine contents in the needles at lower TAW (Table 5 and S5B, S5D and S5F Fig). Likewise, other studies showed increased TAA contents as an indicator of water stress [69,70] and temperature stress [70]. Furthermore, increased arginine concentrations were reported in earlier studies with Douglas-fir seedlings exposed to controlled drought treatments [43]. Arginine is the proteinogenic amino acid with the lowest C/N ratio. Its accumulation under adverse environmental conditions restricting the N demand for growth might be a response to assimilate excess ammonium in a C-efficient way [26–28]. Moreover, arginine is a precursor for proline, nitric oxide and polyamines, which are compounds playing crucial roles in regulating responses to biotic and abiotic stress [71]. The observed increased arginine contents might thus indicate a physiological acclimatization of the trees to the pronounced reduction of photosynthetic CO_2_ assimilation rate and stomatal conductance under low TAW in our experiment [13].

In contrast to our initial hypothesis and to results for pine seedlings [44], total soluble proteins (TSP) did not decrease with decreasing TAW. Our data instead indicate that in Douglas-fir, decreasing contents of structural N rather than TSP serve as a source to maintain a high foliar amino acid content, as also observed in oak leaves [40].

Statistically significant although with respect to effect size small ‘provenance:TAW’ interaction effects were detected for TAA, arginine, and asparagine. In our initial hypothesis, we expected a smaller increase in amino acids in the interior provenance AR as a consequence of constitutively higher levels of N-rich amino acids such as arginine or asparagine. In contrast to this hypothesis, a smaller increase in arginine or asparagine under low TAW was not evident for the provenance AR. Moreover, ‘provenance:TAW’ interaction effects for arginine, asparagine and TAA were quite small with respect to effect size, and ‘provenance:TAW’ interaction effects were missing for glutamate and the other N pools (Table 5), suggesting that the adjustments of N metabolism in response to ‘TAW’ observed here is largely independent from constitutive differences in N pool sizes and composition. These findings illustrate that phenotypic plasticity of N metabolism in response to ‘TAW’ is largely conserved among the Douglas-fir provenances studied here.

### Foliar N status of adult Douglas-fir trees is independent from site-related differences in soil N content

In our statistical analysis we used TAW to estimate soil water availability integrating important site-related factors including temperature, precipitation, latitude, slope and soil type. TAW was generally higher at site S compared to site W and thus was assumed to capture a large fraction of environmental variation between the two sites. In agreement with this assumption, the effect sizes of the generic factor ‘site’ were low for N pools and major amino acids (Table 5). It is intuitive to consider variation in soil N content as the underlying environmental factor accounting for effects of ‘site’ independent from effects of ‘TAW’. However, needle N pools were significantly higher at W compared to S (Fig 3, Table 5 and S5A–S5D Fig), despite significantly higher soil total N and dissolved mineral N contents at S compared to W (Table 3). This finding contrasts previous reports indicating higher leaf N contents in trees grown at sites of higher N availability or in N fertilization experiments [17–19,26,72,73]. Several possibilities might explain these unexpected observations:

1. In forest soils, a high proportion of total N is incorporated in organic macromolecules such as proteins or chitin. Such compounds cannot be taken up directly by the roots of the trees and may lead to a discrepancy between total soil N content and available dissolved soil N content could, therefore, explain the differences between total N in the soil and N abundance in plant tissues [74]. However, both the higher natural ^15^N-abundance and higher levels of inorganic N at S compared to W, do not support this assumption, because they suggest a faster soil N-turnover at S [75] and, consequently, a better N supply with plant available N forms. Furthermore, the lower C / N ratios at S compared to W (Table 3) suggest reduced soil microbial competition for N against plants due to C limitation of heterotrophic soil microbes, thereby leaving more dissolved N for uptake by trees [76].
2. The prevailing environmental conditions, such as the considerably cooler air temperatures at S, could have impaired root N uptake of the trees due to lower soil temperatures and a shorter growing season at S compared to W. This idea is supported by temperature dependencies of N uptake [77] and the observed slower growth of the experimental trees at S compared to W [11]. Moreover, it has been shown that elevated temperatures can cause higher needle N contents in Douglas-fir [78–80]. This is due to increased N uptake as a consequence of improved soil N availability [81,82], which most likely is the result of higher microbial activity in litter and soil organic matter [83,84]. Furthermore, the effect of temperature on root N uptake also depends on soil N status. For instance, N uptake by roots of trees was increased with increasing temperatures at low soil N availability (comparable to site W in our study), but was decreased in N rich soils [85].
3. The different air temperatures at the two sites could have influenced the general N allocation patterns in the trees. Hu et al. [40] found enhanced biosynthesis of proteins and amino acids instead of incorporation into structural components in oak under drought and / or air warming. Effects of N availability on N allocation patterns were also reported by Gezelius and Näsholm [86]who observed high TAA concentrations in shoot, needles, and stems of Scots pine seedlings grown under low N supply, but higher concentrations of free amino acids in the roots of seedlings grown under higher N supply.

Consistent with findings on Scots pine [24,28,87] and Norway spruce [88], in the present study up to 19.8% of the total N in Douglas-fir needles was stored in free amino acids, predominantly as arginine, asparagine and glutamate. At W, arginine dominated the TAA profiles whereas this amino acid was virtually absent in Douglas-fir needles at S. Because arginine is also considered a N storage compound, our results suggest enhanced allocation into the N storage pool at W compared to S.

### Variation of needle N pools among provenances is small compared to variation related to environmental conditions

Based on the assumption of local adaptation to N availability and environmental restrictions to carbon assimilation at the provenances’ native habitats, we hypothesized that Douglas-fir provenances differ in N pool sizes and -composition. Bond et al. [89] reported needle N contents of 12.5±1.3 mg g^-1^ for trees from a stand in the Willamette Valley (Oregon, USA). In our study, total foliar N content varied from 13.0 (AR at S) to 19.7 mg g^-1^ (CR at W). Significant provenance effects on N pools (except structural N), arginine, and glutamate revealed by our study suggest that signatures of local adaptation on N metabolism are detectable in the provenances studied here (Table 5). In contrast to our initial hypothesis, the interior provenance AR did not exhibit the highest levels of TSP, arginine or asparagine. Moreover, the proportion of variance explained by the factor ‘provenance’ is quite low compared to factors capturing environmental variation at the field sites, such as ‘TAW’ and ‘site’ (Table 5). This indicates that genetic differentiation in N metabolism is probably rather limited among the Douglas-fir provenances studied here. A high gene flow among Douglas-fir populations, especially among coastal provenances [89] might contribute to this finding.

Low genetic differentiation in N metabolism was also concluded from a common garden experiment with young *Picea glauca* trees from six seed sources, which did not differ with respect to their needle N content [34]. In contrast, when trees originating from the same six *Picea glauca* seed orchards were studied in a greenhouse experiment, small but significant variation in needle N content was detected, which was negatively correlated with mean growing season air temperature (MGST) at the seed orchard [90].

Raitio and Sarjala [25] reported that free amino acids in needles of Scots pine were more significantly affected by provenances as compared to experimental sites. Two common garden studies with Norway spruce [20] and Scots pine [24] reported increasing needle N concentrations with increasing altitude and decreasing mean annual temperature (MAT) [20] or with increasing latitude [24] of seed source origin. It was concluded that higher needle N concentrations were adaptive since they often contain large quantities of Rubisco [29,30] and are correlated with higher maximum photosynthetic capacity (*A*_max_) [7,20,24,91]. A higher *A*_max_, in turn, might at least partially compensate for environmental restrictions in carbon gain in habitats with low temperatures in higher altitudes or latitudes [20,24]. In our study, no obvious trend of total needle N with latitude, altitude, MAT or MGST was evident (Table 1 and Fig 3). However, we found a trend towards increasing TSP content with increasing MAT: provenance AR originating from the coolest habitat had lowest TSP content, provenances CR and RI had intermediate TSP content, and provenance LA, originating from the warmest native habitat, had highest TSP contents (Table 1 and Fig 3).

### Does needle N content correlate with photosynthetic CO_2_ assimilation rates or growth?

To further assess whether intraspecific variation in needle total N was linked to intraspecific variation in adaptive traits related to carbon gain, we analyzed the correlation of total N content with net photosynthetic CO_2_ assimilation rate (*A*) collected at the same trees by Junker et al. [13]. For this analysis we selected data obtained in July at Schluchsee, when TAW was least restrictive for *A*. In contrast to results for Norway spruce, but in agreement with results for *Picea glauca* [34] no significant correlation between *A* and total N content was detected (r = -0.11, P = 0.683 for 2010; r = -0.09, P = 0.782 for 2011, S6 Fig). These findings do not support the concept that variation in needle N content among Douglas-fir provenances is related to adaptive variation in maximum C assimilation rates. It is known that in conifer needles Rubisco is not only present in a photosynthetically active form, but also a as storage form [92]. Along this line, Ripullone et al. [91] reported decreasing proportions of active Rubisco with increasing leaf N content in Douglas-fir. Thus, rather than being related to concentrations of Rubisco, provenance effects on CO_2_ assimilation rates might be a consequence of variation in CO_2_ diffusion related to stomatal and mesophyll conductance as also concluded from Benomar et al. [24] for field-grown *Picea glauca*.

A complementary study based on the same experiment revealed significant differences in height growth among the provenances analyzed here [11]. Provenance CR showed vigorous height growth across common garden sites, and this was associated with highest total N content (Fig 3). The interior provenance AR which exhibited lowest total N and TSP contents in our study (Fig 3), showed lowest height growth across common garden sites [11]. Both, total N content and TSP, were significantly correlated with tree height (S7 Fig). These findings are in agreement with previous research suggesting a role of needle N content for height growth [93].

## Conclusions

This study reveals that across our study set of four genetically differentiated Douglas-fir provenances, needle N content is independent from soil N content. Small provenance effects suggest that genetic differentiation in N metabolism among the studied provenances is limited. Provenances displayed phenotypic plasticity to decreasing soil water availability by recruiting a largely conserved acclimatization of N metabolism involving a re-allocation of N from structural N to metabolic N. Small or missing interaction effects of ‘provenance’ with soil water availability or ‘site’ indicate that the variation in phenotypic plasticity among provenances is very limited. In the context of our earlier studies on the same experiment [13], our results further reveal that net photosynthetic CO_2_ assimilation rates do not increase with increasing needle N content. Further studies with a set of provenances spanning strong climatic gradients at low geographical and thus phylogenetic distance are needed to test how far these conclusions can be generalized.

## Supporting information

S1 Table Amino acids and ammonia content (μmol g^-1^ DW) in previous year needles of the four provenances (AR, CR, RI and LA) in May and July 2010 (5/10 and 7/10) and 2011 (5/11 and 7/11) at two sites (Wiesloch and Schluchsee). (PDF)

S2 Table Results of sequential regression of N compounds in needles of adult Douglas-fir trees of four provenances on soil water availability and site effects independent from soil water availability. (PDF)

S1 Fig Variable Importance in Projection (VIP) scores of component 1 from PLS_DA shown the importance of factors determining the site related (W, Wiesloch; S, Schluchsee) nitrogen partitioning patterns in 2010 (A) and 2011 (B). (PDF)

S2 Fig Variable Importance in Projection (VIP) scores of component 1 from PLS_DA shown the importance determining the seasons related nitrogen partitioning patterns in May (5/10) and July (7/10) 2010 (A, B) and 2011(May, 5/11 and July, 7/11) (C, D) at Wiesloch (W) and Schluchsee (S) respectively. (PDF)

S3 Fig Provenance-related patterns of nitrogen partitioning in previous year needles of the four Douglas-fir provenances sampled in 2010 (AR, Salmon Arm; CR, Conrad Creek; LA, Cameron Lake; RI, Santiam River). Clustering was revealed by score plots of partial least squares–discriminant analyses.
(PDF)

S4 Fig Provenance-related patterns of nitrogen partitioning in previous year needles of the four Douglas-fir provenances sampled in 2011 (AR, Salmon Arm; CR, Conrad Creek; LA, Cameron Lake; RI, Santiam River). Clustering was revealed by score plots of partial least squares–discriminant analyses.
(PDF)

S5 Fig Nitrogen (N) pools and compounds in needles of adult Douglas-fir trees of four provenances grown on two field sites in south-western Germany (Wiesloch and Schluchsee). Data are plotted over experimental and environmental factors identified as significant predictors (for details of the statistical analysis see Material and methods; data are plotted on the scale used in statistical analysis).
(PDF)

S6 Fig Correlation between needle total N pools in needles of adult Douglas-fir trees of four provenances grown on field site ‘Schluchsee’ in south-western Germany with net photosynthetic assimilation rate (*A*) measured on the same trees in July 2010 and 2011. (PDF)

S7 Fig Correlation between needle total nitrogen (N) pools in needles of adult Douglas-fir trees of four provenances with tree height. Trees were grown on two field sites in south-western Germany.
(PDF)
