# Supplementary material for: Foliar nitrogen metabolism of adult Douglas-fir trees is affected by soil water availability and varies little among provenances
Source: PLoS One. 2018 Mar 22;13(3):e0194684. doi: 10.1371/journal.pone.0194684 (PMC5864041; doi:10.1371/journal.pone.0194684)
Supplement: S1 Table — (PDF) [file pone.0194684.s001.pdf]

Supporting Information

Foliar nitrogen metabolism of adult Douglas-fir trees is affected by soil water availability and varies little among provenances

Baoguo Du, Jürgen Kreuzwieser, Michael Dannenmann, Laura V. Junker, Anita Kleiber, Moritz Hess, Kirstin Jansen, Monika Eiblmeier, Arthur Gessler, Ulrich Kohnle, Ingo Ensminger, Heinz Rennenberg, Henning Wildhagen*

* Correspondence: Henning Wildhagen, HAWK University of Applied Sciences and Arts Hildesheim/Holzminden/Göttingen, Faculty of Resource Management, Büsgenweg 1A, 37077 Göttingen, Germany. Email: henning.wildhagen@hawk.de

S1 Table

Amino acids and ammonia content (μmol g^{-1} DW) in previous year needles of the four provenances (AR, Salmon Arm; CR, Conrad Creek; LA, Cameron Lake; RI, Santiam River) in May and July 2010 (5/10 and 7/10) and 2011 (5/11 and 7/11) at two sites (Wiesloch and Schluchsee). Data shown mean ±SE. ND: not detectable, more than 3 of each 6 replicates were below detection limit.

	**Time**	**Wiesloch**				**Schluchsee**			
	**(M/Y)**	AR	CR	LA	RI	AR	CR	LA	RI
Histidine	5/10	0.19±0.03	0.21±0.06	0.15±0.02	0.14±0.03	ND	0.44±0.15	ND	ND
	7/10	0.22±0.03	0.53±0.15	0.27±0.07	0.23±0.05	ND	ND	ND	ND
	5/11	0.56±0.09	0.38±0.05	0.40±0.06	0.42±0.09	0.18±0.11	ND	ND	ND
	7/11	ND	ND	ND	ND	ND	ND	ND	ND
NH_3	5/10	1.66±0.33	1.45±0.26	1.12±0.60	1.94±0.26	1.77±0.21	1.71±0.22	1.97±0.26	1.89±0.25
	7/10	3.24±0.22	3.08±0.18	3.16±0.16	3.79±0.63	1.14±0.17	1.80±0.44	0.69±0.14	ND
	5/11	1.04±0.15	1.06±0.20	2.11±0.55	2.06±0.51	2.64±0.15	3.55±0.77	2.11±0.58	3.22±0.77
	7/11	2.73±0.12	3.04±0.24	2.38±0.14	2.39±0.32	0.19±0.04	0.24±0.05	0.31±0.05	0.28±0.11
Glycine	5/10	0.03±0.01	0.07±0.02	0.06±0.02	ND	0.12±0.01	0.11±0.01	0.13±0.01	0.13±0.01

	Time (M/Y)	Wiesloch				Schluchsee			
		AR	CR	LA	RI	AR	CR	LA	RI
	7/10	ND	ND	ND	ND	ND	ND	ND	ND
	5/11	ND	ND	ND	ND	0.19±0.01	ND	0.18±0.01	ND
	7/11	ND	ND	ND	ND	ND	ND	ND	ND
Cysteine	5/10	0.18±0.03	0.27±0.12	0.16±0.01	0.12±0.01	0.17±0.01	0.30±0.09	0.16±0.01	0.17±0.01
	7/10	ND	ND	ND	ND	0.21±0.09	0.32±0.04	0.09±0.06	ND
	5/11	ND	ND	ND	ND	0.49±0.04	ND	ND	ND
	7/11	0.25±0.04	0.20±0.02	0.21±0.01	ND	0.19±0.06	ND	ND	ND
Alanine	5/10	0.60±0.12	0.60±0.06	0.64±0.03	0.54±0.03	0.70±0.09	0.67±0.05	0.64±0.04	0.63±0.04
	7/10	0.71±0.15	0.28±0.04	0.42±0.18	0.25±0.05	0.27±0.04	0.38±0.06	0.31±0.03	0.26±0.07
	5/11	0.74±0.05	0.81±0.13	0.69±0.05	0.86±0.09	0.80±0.09	1.01±0.21	0.84±0.10	0.81±0.11
	7/11	0.78±0.05	0.81±0.09	0.63±0.05	0.72±0.07	0.40±0.02	0.51±0.06	0.49±0.03	0.46±0.04
Valine	5/10	0.16±0.06	0.19±0.10	0.22±0.07	0.14±0.03	0.17±0.02	0.22±0.01	0.21±0.01	0.20±0.01
	7/10	ND	ND	ND	ND	0.06±0.01	0.05±0.01	0.06±0.01	0.05±0.01
	5/11	0.19±0.01	0.21±0.03	0.18±0.02	0.21±0.03	0.34±0.04	ND	0.36±0.02	ND

	Time (M/Y)	Wiesloch				Schluchsee			
		AR	CR	LA	RI	AR	CR	LA	RI
	7/11	0.21±0.05	0.19±0.02	0.19±0.02	0.20±0.02	ND	0.12±0.02	ND	ND
Isoleucine	5/10	0.09±0.04	0.09±0.02	0.16±0.04	0.21±0.02	0.28±0.03	0.35±0.03	0.34±0.02	0.32±0.02
	7/10	0.03±0.02	0.06±0.02	0.08±0.02	0.13±0.04	0.23±0.03	0.26±0.03	0.17±0.03	0.23±0.04
	5/11	0.42±0.04	0.42±0.06	0.38±0.05	0.49±0.06	0.27±0.02	ND	0.25±0.02	ND
	7/11	0.26±0.06	0.23±0.06	0.19±0.03	0.28±0.06	0.19±0.02	0.37±0.09	0.38±0.06	ND
Leucine	5/10	0.21±0.05	0.21±0.04	0.25±0.03	0.27±0.02	0.19±0.01	0.27±0.04	0.24±0.02	0.20±0.03
	7/10	0.20±0.03	0.21±0.04	0.19±0.03	0.27±0.04	0.29±0.08	0.24±0.06	0.13±0.01	0.16±0.03
	5/11	0.55±0.06	0.54±0.10	0.35±0.07	0.45±0.03	0.28±0.03	0.11±0.04	0.27±0.02	ND
	7/11	0.31±0.03	0.30±0.03	0.26±0.02	0.35±0.02	0.36±0.03	0.44±0.08	0.46±0.08	0.35±0.02
Tyrosine	5/10	3.09±0.77	1.13±0.49	2.53±0.53	2.38±0.20	1.23±0.15	0.95±0.17	1.31±0.1	1.30±0.08
	7/10	2.17±0.41	1.53±0.38	1.57±0.23	1.26±0.32	0.22±0.02	0.19±0.01	0.20±0.03	0.19±0.01
	5/11	1.55±0.31	0.65±0.14	0.83±0.13	0.62±0.14	0.58±0.04	0.59±0.07	0.61±0.04	0.58±0.0
	7/11	0.35±0.10	0.44±0.10	0.37±0.13	0.36±0.06	0.52±0.03	0.74±0.18	0.45±0.05	0.76±0.09
Phenylalanine	5/10	0.19±0.02	0.17±0.02	0.19±0.02	0.15±0.02	0.21±0.01	0.24±0.01	0.22±0.01	0.24±0.01

	Time (M/Y)	Wiesloch				Schluchsee			
		AR	CR	LA	RI	AR	CR	LA	RI
	7/10	0.13±0.01	0.20±0.05	0.13±0.01	0.10±0.01	0.12±0.01	0.08±0.01	0.10±0.01	0.08±0.01
	5/11	ND	0.20±0.02	0.26±0.02	ND	0.42±0.02	ND	0.38±0.01	0.01±0.01
	7/11	0.23±0.03	0.23±0.02	0.20±0.01	0.18±0.01	ND	0.12±0.02	ND	ND
Tryptophan	5/10	0.12±0.05	0.04±0.01	0.15±0.04	0.09±0.04	0.22±0.05	0.29±0.05	0.26±0.04	0.27±0.04
	7/10	0.37±0.05	0.81±0.18	0.40±0.07	0.30±0.04	0.13±0.01	0.16±0.02	0.13±0.01	0.20±0.04
	5/11	0.54±0.09	0.36±0.07	0.30±0.03	0.33±0.05	0.41±0.05	ND	0.39±0.04	ND
	7/11	0.31±0.06	0.44±0.11	0.38±0.10	0.42±0.12	0.19±0.03	0.25±0.05	0.25±0.06	0.21±0.02
Glutamine	5/10	0.57±0.13	0.90±0.14	1.00±0.30	0.78±0.20	0.41±0.11	0.48±0.05	0.78±0.17	0.89±0.19
	7/10	0.35±0.04	0.30±0.09	0.28±0.02	0.27±0.04	ND	ND	0.22±0.03	ND
	5/11	0.44±0.05	0.37±0.10	0.34±0.03	0.53±0.05	0.45±0.08	0.90±0.57	0.54±0.13	0.32±0.09
	7/11	0.33±0.06	0.22±0.03	ND	0.27±0.06	0.26±0.02	0.26±0.04	0.25±0.04	0.25±0.05
Proline	5/10	1.69±0.51	1.59±0.34	1.03±0.33	1.02±0.28	1.68±0.46	1.28±0.25	1.84±0.47	1.73±0.39
	7/10	1.93±0.68	1.41±0.47	0.84±0.13	0.83±0.17	0.28±0.04	0.29±0.04	0.24±0.03	0.25±0.05
	5/11	1.57±0.35	0.88±0.20	0.79±0.17	1.31±0.41	1.24±0.30	1.50±0.45	1.00±0.16	1.32±0.19

	Time (M/Y)	Wiesloch				Schluchsee			
		AR	CR	LA	RI	AR	CR	LA	RI
	7/11	0.59±0.10	0.48±0.04	0.45±0.04	0.37±0.05	0.25±0.02	0.36±0.04	0.35±0.04	0.27±0.02
Ornithine	5/10	ND	0.73±0.32	ND	ND	0.13±0.01	1.50±0.56	0.13±0.01	0.19±0.02
	7/10	ND	0.67±0.27	ND	ND	0.07±0.01	0.14±0.04	0.06±0.01	ND
	5/11	0.79±0.22	ND	0.07±0.03	0.06±0.02	0.37±0.03	ND	0.35±0.01	ND
	7/11	0.62±0.14	ND	ND	ND	0.02±0.01	0.72±0.28	0.56±0.28	ND
Citrulline	5/10	ND	ND	ND	ND	0.35±0.04	0.38±0.02	0.30±0.02	0.35±0.03
	7/10	ND	ND	ND	ND	ND	ND	ND	ND
	5/11	ND	ND	ND	ND	0.31±0.04	ND	0.24±0.02	ND
	7/11	ND	ND	0.69±0.13	ND	ND	ND	ND	ND
Aspartic acid	5/10	1.08±0.19	1.44±0.20	1.19±0.31	1.59±0.24	0.83±0.07	1.06±0.09	1.34±0.23	1.11±0.14
	7/10	1.04±0.07	1.07±0.10	1.19±0.08	1.16±0.03	0.72±0.11	0.65±0.14	0.86±0.11	0.54±0.15
	5/11	1.14±0.11	0.99±0.12	1.21±0.18	1.36±0.15	1.16±0.11	1.19±0.11	1.49±0.28	1.43±0.16
	7/11	1.10±0.07	1.19±0.12	0.96±0.07	1.20±0.03	0.53±0.04	0.65±0.08	0.89±0.14	0.66±0.07
Methionine	5/10	0.04±0.01	0.03±0.01	0.06±0.02	0.05±0.01	0.23±0.02	0.26±0.01	0.26±0.01	0.28±0.01

	Time (M/Y)	Wiesloch				Schluchsee			
		AR	CR	LA	RI	AR	CR	LA	RI
	7/10	0.08±0.01	0.11±0.02	0.06±0.01	0.09±0.01	0.14±0.02	0.19±0.02	0.21±0.01	0.18±0.01
	5/11	0.21±0.02	0.29±0.06	0.25±0.04	0.21±0.02	0.35±0.02	ND	0.36±0.01	0.03±0.01
	7/11	0.15±0.01	0.18±0.01	0.30±0.08	0.20±0.01	ND	0.13±0.01	0.20±0.04	0.20±0.03
Threonine	5/10	0.06±0.01	0.08±0.01	0.09±0.01	0.08±0.01	0.21±0.01	0.24±0.01	0.24±0.01	0.24±0.01
	7/10	0.13±0.01	0.14±0.01	0.13±0.02	0.13±0.01	ND	ND	ND	ND
	5/11	ND	ND	ND	0.22±0.03	0.37±0.02	ND	0.38±0.02	ND
	7/11	ND	ND	ND	ND	ND	ND	ND	ND
Lysine	5/10	ND	0.76±0.29	ND	ND	0.24±0.03	1.56±0.54	0.20±0.04	0.11±0.02
	7/10	0.53±0.17	1.37±0.47	0.73±0.21	0.71±0.35	0.28±0.08	0.13±0.06	0.36±0.06	0.15±0.06
	5/11	ND	0.65±0.11	ND	0.48±0.11	0.80±0.06	ND	0.62±0.03	ND
	7/11	0.77±0.25	0.71±0.31	ND	ND	0.17±0.01	1.24±0.33	0.96±0.28	0.25±0.03
γ–aminobutyric acid	5/10	ND	0.10±0.01	ND	ND	0.22±0.04	ND	ND	0.24±0.03
	7/10	0.04±0.01	0.07±0.02	0.12±0.04	ND	0.37±0.06	0.28±0.07	0.34±0.08	0.31±0.07
	5/11	ND	ND	ND	ND	0.09±0.01	ND	0.09±0.01	ND

	Time (M/Y)	Wiesloch				Schluchsee			
		AR	CR	LA	RI	AR	CR	LA	RI
	7/11	ND	ND	ND	ND	ND	ND	ND	ND
α-aminobutyric acid	5/10	0.45±0.08	0.56±0.09	0.55±0.09	0.66±0.07	0.35±0.03	0.35±0.01	0.35±0.02	0.37±0.01
	7/10	ND	ND	ND	ND	ND	ND	ND	ND
	5/11	0.23±0.03	0.22±0.05	0.18±0.01	0.20±0.02	0.74±0.05	0.17±0.02	0.83±0.08	0.12±0.03
	7/11	0.13±0.01	0.14±0.01	0.12±0.01	0.12±0.01	0.19±0.03	0.17±0.02	0.20±0.03	0.16±0.01
