# Supplementary material for: Foliar nitrogen metabolism of adult Douglas-fir trees is affected by soil water availability and varies little among provenances
Source: PLoS One. 2018 Mar 22;13(3):e0194684. doi: 10.1371/journal.pone.0194684 (PMC5864041; doi:10.1371/journal.pone.0194684)
Supplement: S2 Table — (PDF) [file pone.0194684.s002.pdf]

Supporting Information

Foliar nitrogen metabolism of adult Douglas-fir trees is affected by soil water availability and varies little among provenances

Baoguo Du, Jürgen Kreuzwieser, Michael Dannenmann, Laura V. Junker, Anita Kleiber, Moritz Hess, Kirstin Jansen, Monika Eiblmeier, Arthur Gessler, Ulrich Kohnle, Ingo Ensminger, Heinz Rennenberg, Henning Wildhagen*

* Correspondence: Henning Wildhagen, HAWK University of Applied Sciences and Arts Hildesheim/Holzminden/Göttingen, Faculty of Resource Management, Büsgenweg 1A, 37077 Göttingen, Germany. Email: henning.wildhagen@hawk.de

S2 Table

Results of regression of N compounds and pools in needles of adult Douglas-fir trees of four provenances on soil water availability and site effects. N compounds were determined in previous year needles of four provenances (AR, Salmon Arm; CR, Conrad Creek; LA, Cameron Lake; RI, Santiam River) in May and July 2010 (5/10 and 7/10) and 2011 (5/11 and 7/11) at two sites in southwestern Germany (Wiesloch and Schluchsee).

		Sequential regression			
**Dependent variable**	**Independent regression (r^2 for 'TAW' / r^2 for 'site')**	**Explanatory variable**	**P-value**	**Variance proportion**	**r^2/r^2_{adj}**
Total N	0.23 / 0.28	'TAW'	<0.001	0.23	0.30/0.29
		resid('site')	<0.001	0.06	
Total soluble protein	0.24 / 0.08	'TAW'	<0.001	0.25	0.28/0.27
		resid('site')	0.005	0.03	
Structural N	0.06 / 0.001	'TAW'	<0.001	0.06	0.13/0.12
		resid('site')	<0.001	0.07	
Total amino acids	0.24 / 0.23	'TAW'	<0.001	0.29	0.32/0.31
		resid('site')	0.002	0.04	
Asparagine	0.04 / 0.02	'TAW'	0.003	0.05	0.05/0.03
		resid('site')	0.97	<0.001	
Arginine	0.20 / 0.18	'TAW'	<0.001	0.41	0.51/0.50
		resid('site')	<0.001	0.09	
Glutamate	0.27 / 0.17	'TAW'	<0.001	0.27	0.27/0.26
		resid('site')	0.999	<0.001	

In a first step ("Independent regression"), N compounds/pools were regressed separately on 'TAW' and 'site'. For all N compounds/pools except total N, 'TAW' explained more variation compared to 'site'. We considered 'TAW' as the most direct and obvious indicator for water availability and thus regressed in a second step ("Sequential regression") 'site' on 'TAW' ('site' ~ 'TAW') using a generalized linear model (R function 'glm', binomial family, logit link). The residuals of this regression (resid('site')) are orthogonal to 'TAW' are thus considered to represent site effects not directly related to water availability. We then modelled (function 'lm') N compounds/pools as functions of 'TAW' and the residuals (on response scale) of 'site' ~ 'TAW': N compound ~ 'TAW' + resid('site').
