# Supplementary material for: Foliar nitrogen metabolism of adult Douglas-fir trees is affected by soil water availability and varies little among provenances
Source: PLoS One. 2018 Mar 22;13(3):e0194684. doi: 10.1371/journal.pone.0194684 (PMC5864041; doi:10.1371/journal.pone.0194684)

Supporting Information

Foliar nitrogen metabolism of adult Douglas-fir trees is affected by soil water availability and varies little among provenances

Baoguo Du, Jürgen Kreuzwieser, Michael Dannenmann, Laura V. Junker, Anita Kleiber, Moritz Hess, Kirstin Jansen, Monika Eiblmeier, Arthur Gessler, Ulrich Kohnle, Ingo Ensminger, Heinz Rennenberg, Henning Wildhagen*

* Correspondence: Henning Wildhagen, HAWK University of Applied Sciences and Arts Hildesheim/Holzminden/Göttingen, Faculty of Resource Management, Büsgenweg 1A, 37077 Göttingen, Germany. Email: henning.wildhagen@hawk.de

S1 Figure

Variable Importance in Projection (VIP) scores of component 1 from PLS-DA shows the importance of factors determining the site related (W, Wiesloch; S, Schluchsee) nitrogen partitioning patterns in 2010 (A) and 2011 (B). The color code indicates the increasing abundance of the features analyzed by color change from green to red. S. protein, soluble protein; TAA, total amino acids. AR, Salmon Arm; CR, Conrad Creek; LA, Cameron Lake; RI, Santiam River.

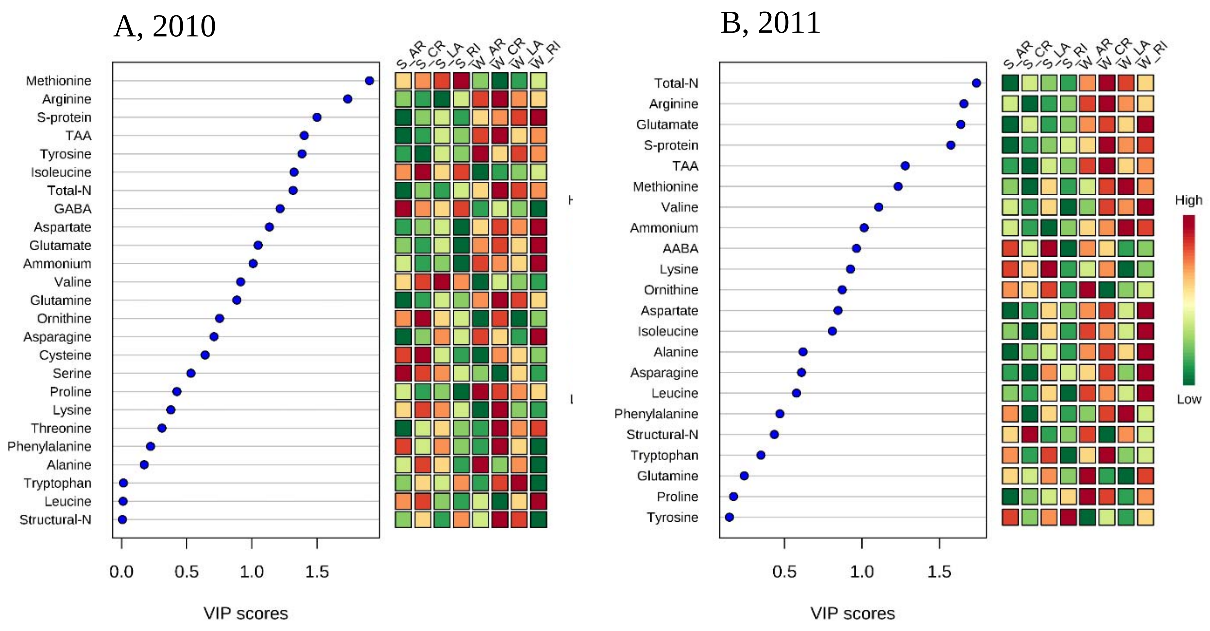

Supplement: S1 Fig — (PDF) [file pone.0194684.s003.pdf]
