# Supplementary material for: Foliar nitrogen metabolism of adult Douglas-fir trees is affected by soil water availability and varies little among provenances
Source: PLoS One. 2018 Mar 22;13(3):e0194684. doi: 10.1371/journal.pone.0194684 (PMC5864041; doi:10.1371/journal.pone.0194684)

Supporting Information

Foliar nitrogen metabolism of adult Douglas-fir trees is affected by soil water availability and varies little among provenances

Baoguo Du, Jürgen Kreuzwieser, Michael Dannenmann, Laura V. Junker, Anita Kleiber, Moritz Hess, Kirstin Jansen, Monika Eiblmeier, Arthur Gessler, Ulrich Kohnle, Ingo Ensminger, Heinz Rennenberg, Henning Wildhagen*

* Correspondence: Henning Wildhagen, HAWK University of Applied Sciences and Arts Hildesheim/Holzminden/Göttingen, Faculty of Resource Management, Büsgenweg 1A, 37077 Göttingen, Germany. Email: henning.wildhagen@hawk.de

S2 Figure

Variable Importance in Projection (VIP) scores of component 1 from PLS-DA shown the importance determining the seasons related nitrogen partitioning patterns in May (5/10) and July (7/10) 2010 (A, B) and 2011(May, 5/11 and July, 7/11) (C, D) at Wiesloch (W) and Schluchsee (S) respectively. Color code, from green to red indicates the increasing abundance of the features analyzed. S. protein, soluble protein; TAA, total amino acids.

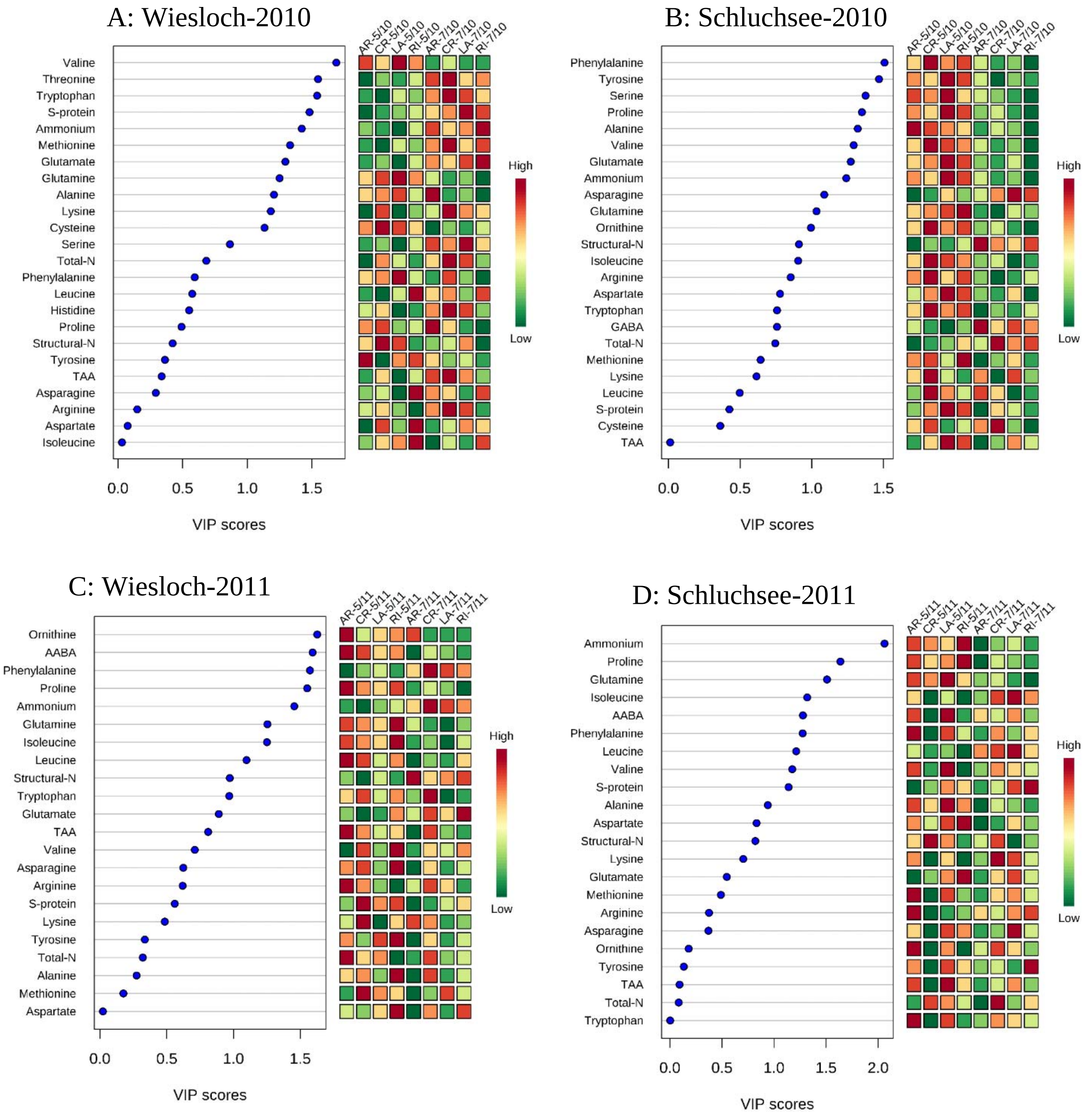

Supplement: S2 Fig — (PDF) [file pone.0194684.s004.pdf]
