# Supplementary material for: Foliar nitrogen metabolism of adult Douglas-fir trees is affected by soil water availability and varies little among provenances
Source: PLoS One. 2018 Mar 22;13(3):e0194684. doi: 10.1371/journal.pone.0194684 (PMC5864041; doi:10.1371/journal.pone.0194684)

Supporting Information

Foliar nitrogen metabolism of adult Douglas-fir trees is affected by soil water availability and varies little among provenances

Baoguo Du, Jürgen Kreuzwieser, Michael Dannenmann, Laura V. Junker, Anita Kleiber, Moritz Hess, Kirstin Jansen, Monika Eiblmeier, Arthur Gessler, Ulrich Kohnle, Ingo Ensminger, Heinz Rennenberg, Henning Wildhagen*

* Correspondence: Henning Wildhagen, HAWK University of Applied Sciences and Arts Hildesheim/Holzminden/Göttingen, Faculty of Resource Management, Büsgenweg 1A, 37077 Göttingen, Germany. Email: henning.wildhagen@hawk.de

S4 Figure

Provenance-related patterns of nitrogen partitioning in previous year needles of the four provenances (AR, Salmon Arm; CR, Conrad Creek; LA, Cameron Lake; RI, Santiam River) revealed by PLS-DA score plots. A and B shown patterns at Wiesloch (W) in May and July 2011 respectively, C and D shown patterns at Schluchsee (S) in May and July 2011 respectively). Semi-transparent shadings indicate 95% confidence regions.

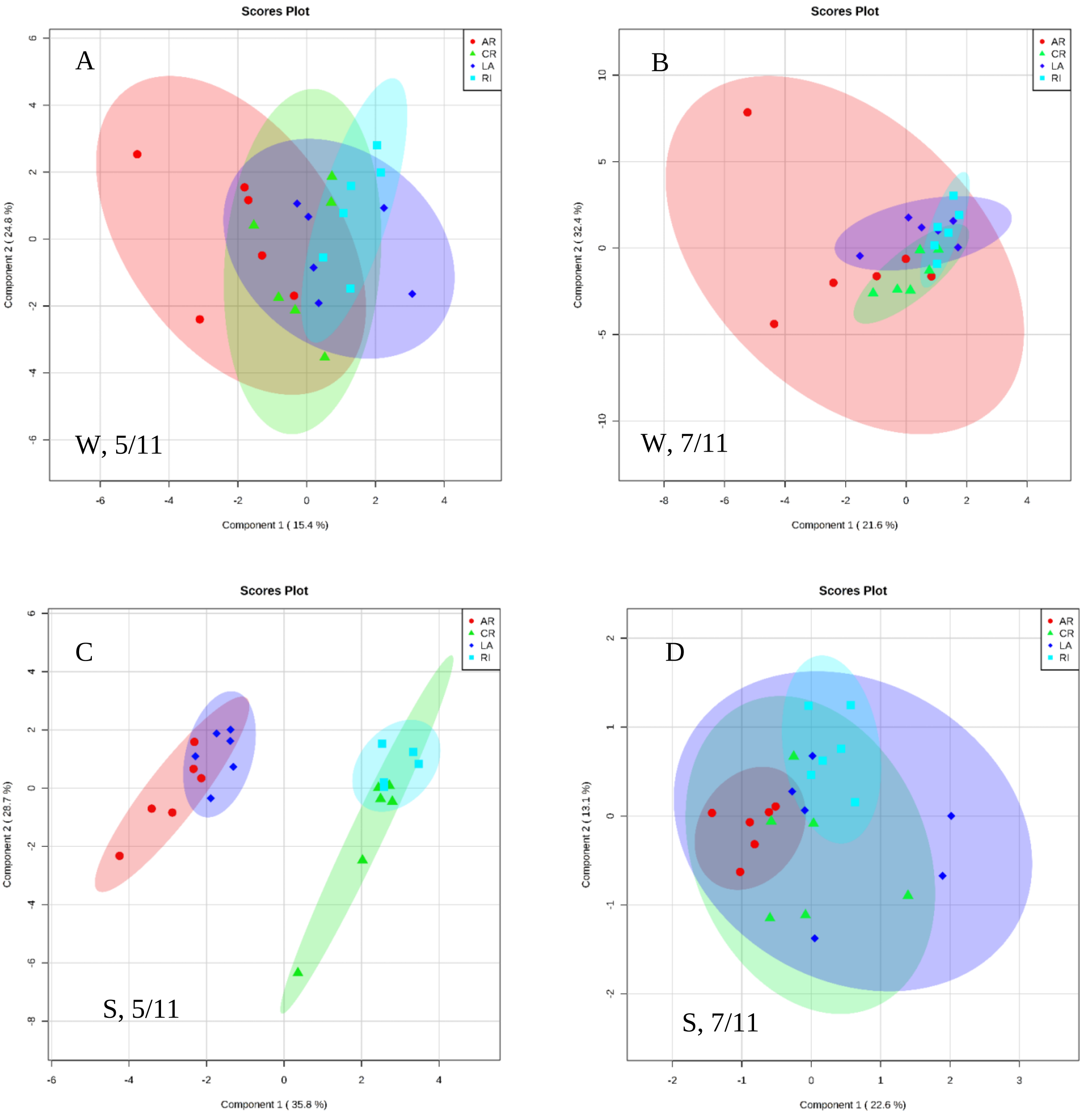

Supplement: S4 Fig — Clustering was revealed by score plots of partial least squares–discriminant analyses. (PDF) [file pone.0194684.s006.pdf]
