# Supplementary material for: Foliar nitrogen metabolism of adult Douglas-fir trees is affected by soil water availability and varies little among provenances
Source: PLoS One. 2018 Mar 22;13(3):e0194684. doi: 10.1371/journal.pone.0194684 (PMC5864041; doi:10.1371/journal.pone.0194684)
Supplement: S5 Fig — Data are plotted over experimental and environmental factors identified as significant predictors (for details of the statistical analysis see Material and methods; data are plotted on the scale used in statistical analysis). (PDF) [file pone.0194684.s007.pdf]

Supporting Information

Foliar nitrogen metabolism of adult Douglas-fir trees is affected by soil water availability and varies little among provenances

Baoguo Du, Jürgen Kreuzwieser, Michael Dannenmann, Laura V. Junker, Anita Kleiber, Moritz Hess, Kirstin Jansen, Monika Eiblmeier, Arthur Gessler, Ulrich Kohnle, Ingo Ensminger, Heinz Rennenberg, Henning Wildhagen*

* Correspondence: Henning Wildhagen, HAWK University of Applied Sciences and Arts Hildesheim/Holzminden/Göttingen, Faculty of Resource Management, Büsgenweg 1A, 37077 Göttingen, Germany. Email: henning.wildhagen@hawk.de

S5 Figure

Nitrogen (N) pools and compounds in needles of adult Douglas-fir trees of four provenances (AR, Salmon Arm; CR, Conrad Creek; LA, Cameron Lake; RI, Santiam River) grown on two field sites in south-western Germany (Wiesloch and Schluchsee). Data are plotted over experimental and environmental factors identified as significant predictors (for details of the statistical analysis see Material and Methods; data are plotted on the scale used in statistical analysis). A) Total N content, B) Total soluble protein content (sqrt: data were square-root transformed), C) Total amino acids (log2: data were log2-transformed), D) Structural N, E) Asparagine concentrations, F) Arginine concentrations, G) Glutamate concentrations. TAW: total available soil water. Red data points in scatter plots represent site Schluchsee, black data points in scatter plots represent site Wiesloch.

A

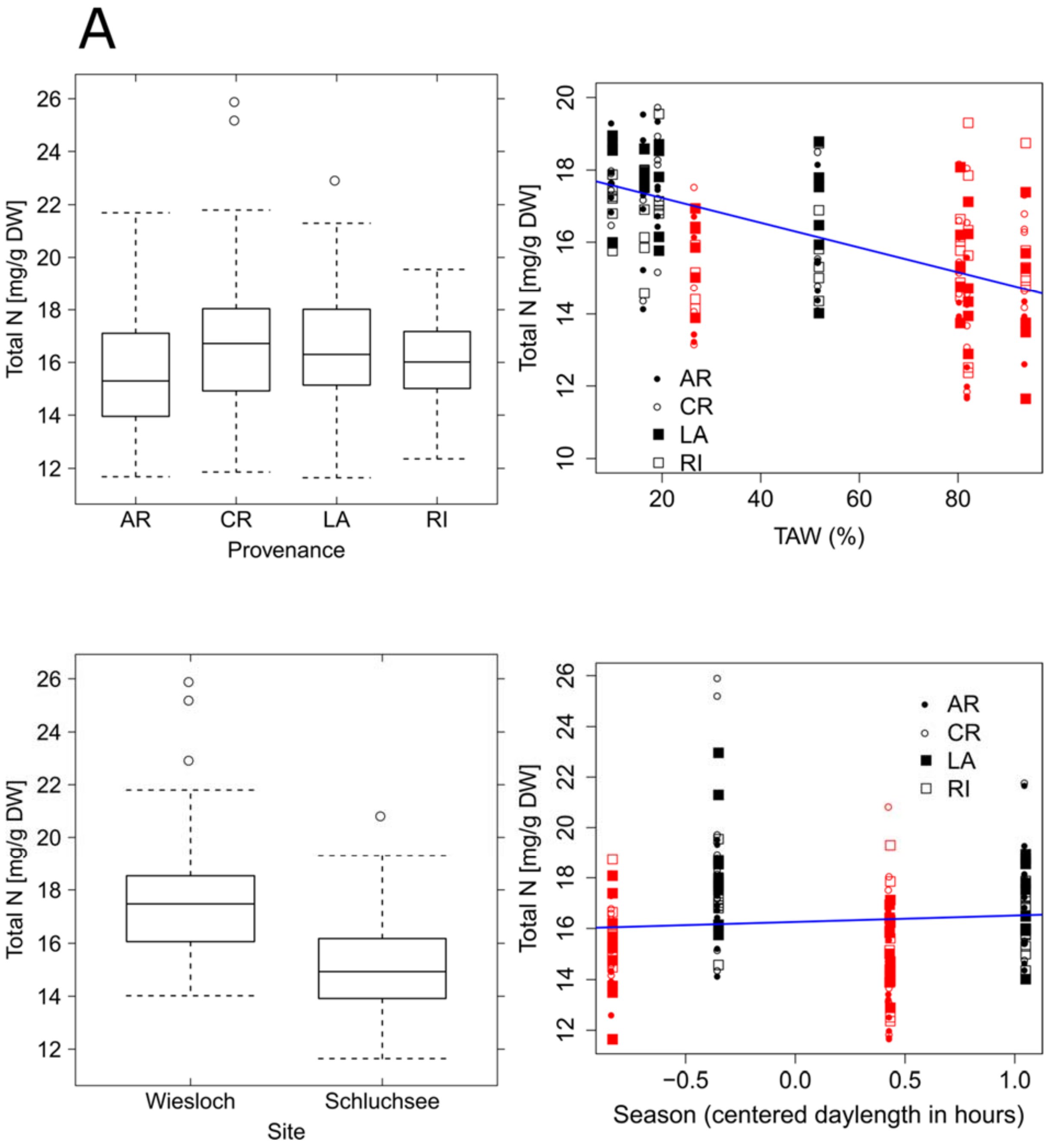
Total N [mg/g DW]
26
24
22
20
18
16
14
12
AR
CR
LA
RI
Provenance
Total N [mg/g DW]
20
18
16
14
12
10
20
40
60
80
TAW (%)
AR
CR
LA
RI
Total N [mg/g DW]
26
24
22
20
18
16
14
12
Wiesloch
Schluchsee
Site
Total N [mg/g DW]
26
24
22
20
18
16
14
12
−0.5
0.0
0.5
1.0
Season (centered daylength in hours)
AR
CR
LA
RI

Figure S5 A

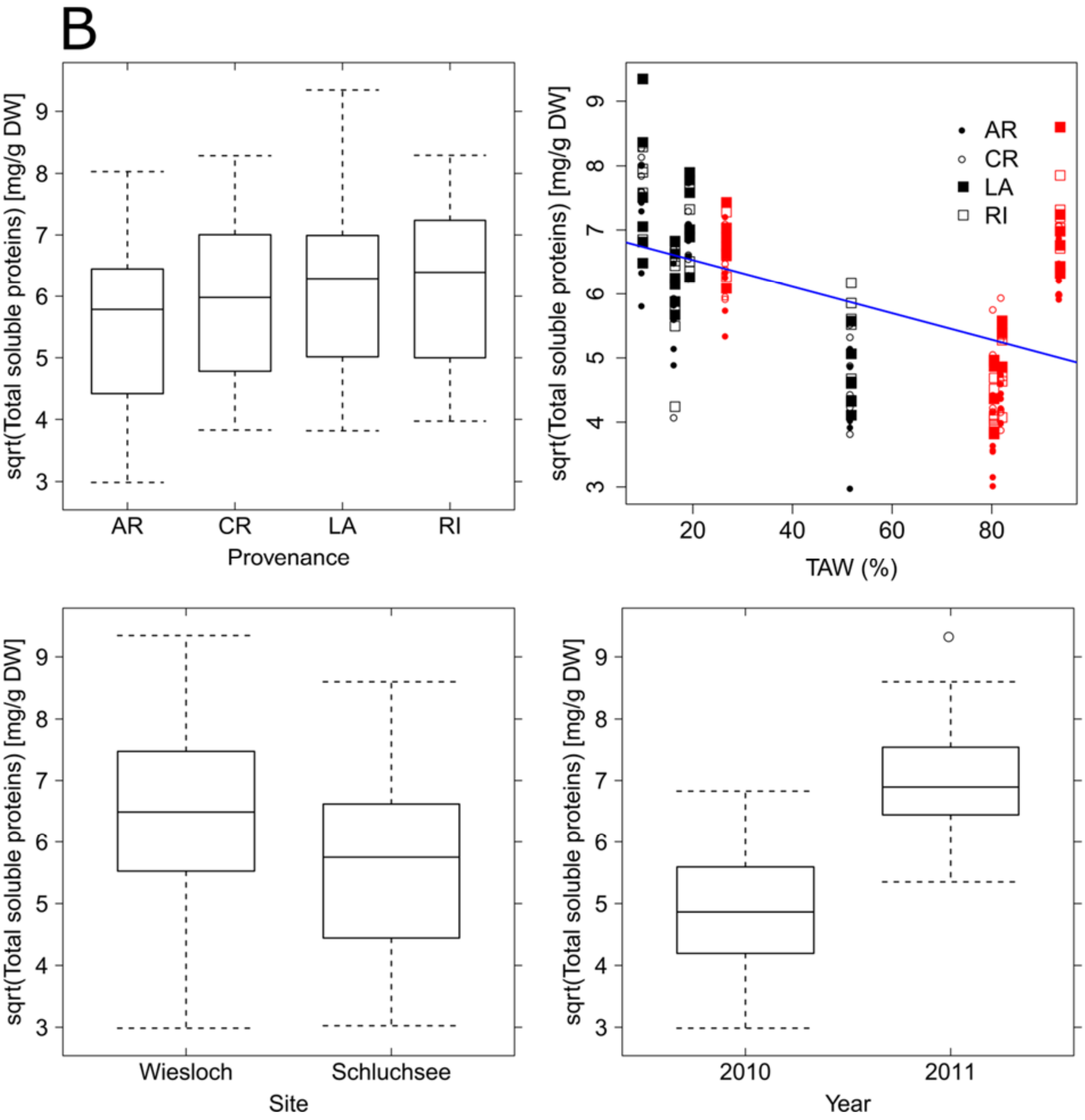
B
sqrt(Total soluble proteins) [mg/g DW]
AR
CR
LA
RI
Provenance
TAW (%)
Wiesloch
Schluchsee
Site
2010
2011
Year

Figure S5 B

C

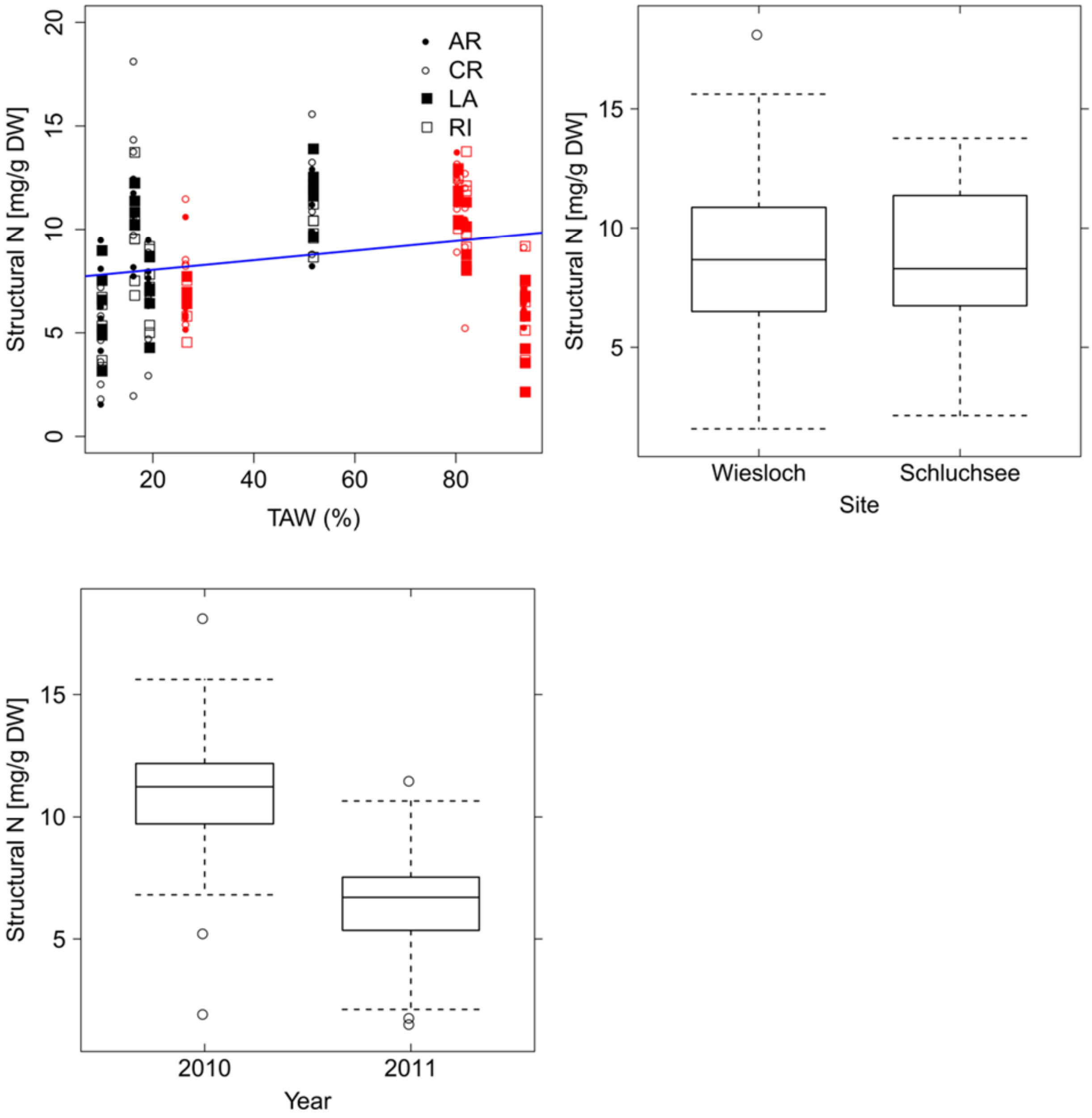
AR
CR
LA
RI
Structural N [mg/g DW]
TAW (%)
0
5
10
15
20
40
60
80
Structural N [mg/g DW]
Wiesloch
Schluchsee
Site
Structural N [mg/g DW]
2010
2011
Year

Figure S5 C

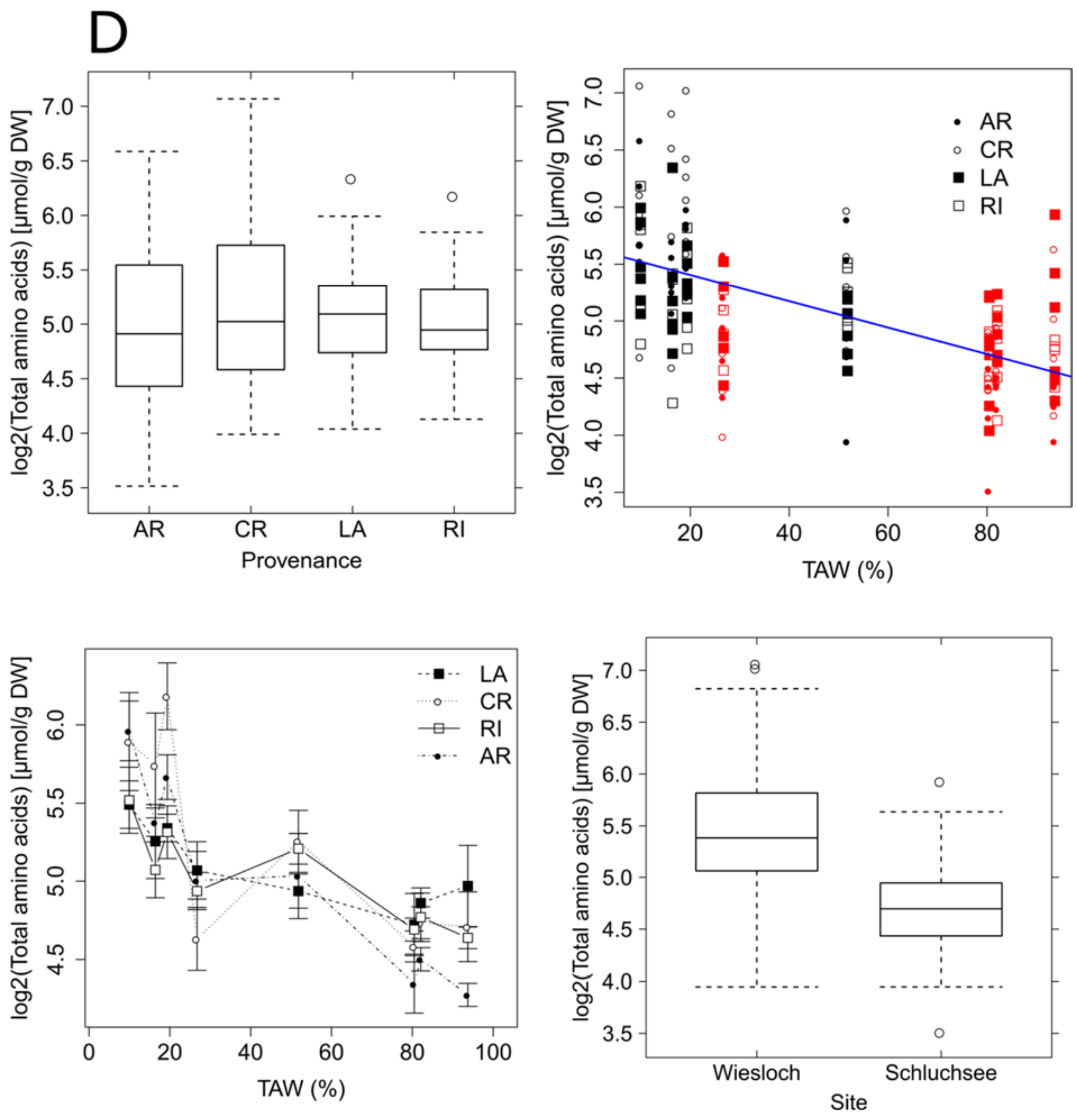

D
log2(Total amino acids) [µmol/g DW]
Provenance
AR
CR
LA
RI
TAW (%)
Wiesloch
Schluchsee
Site

Figure S5 D

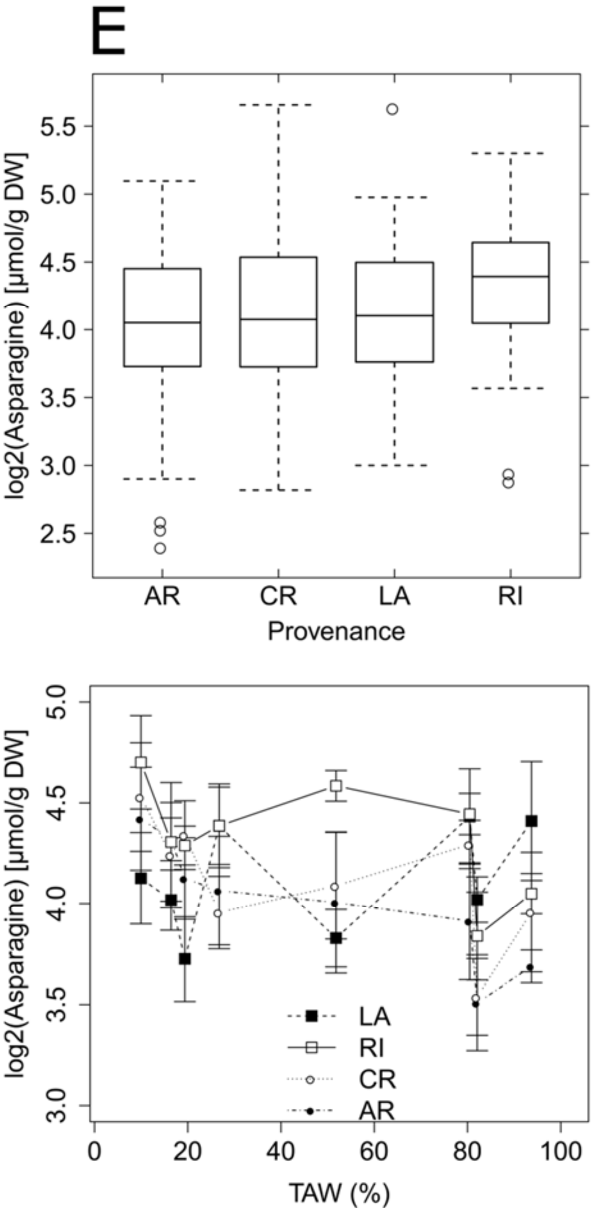
E
log2(Asparagine) [µmol/g DW]
5.5
5.0
4.5
4.0
3.5
3.0
2.5
AR
CR
LA
RI
Provenance
AR
CR
LA
RI
20
40
60
80
TAW (%)
LA
RI
CR
AR
0
20
40
60
80
100
TAW (%)

Figure S5 E

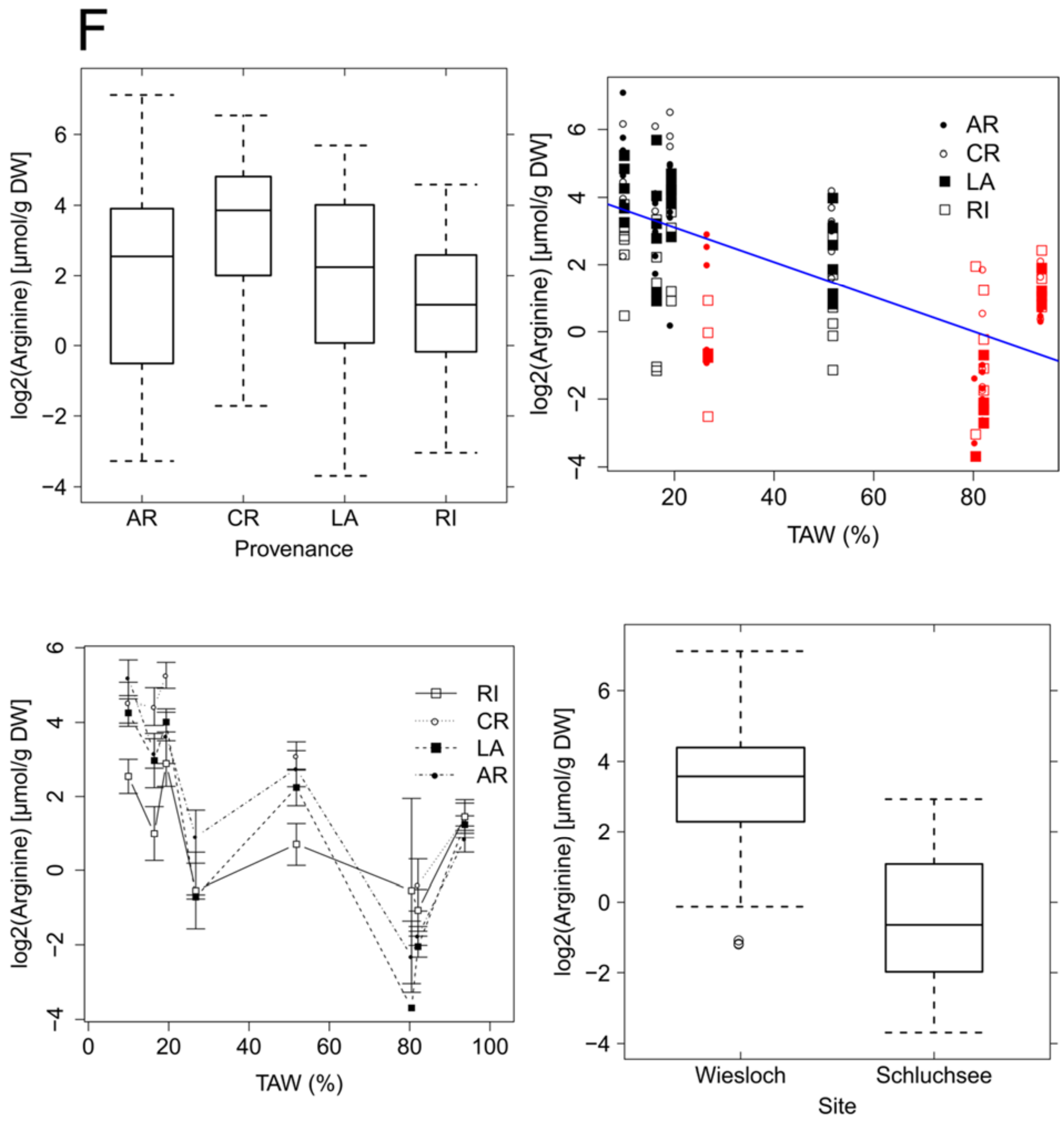
F
log2(Arginine) [µmol/g DW]
AR
CR
LA
RI
Provenance
TAW (%)
Wiesloch
Schluchsee
Site

Figure S5 F

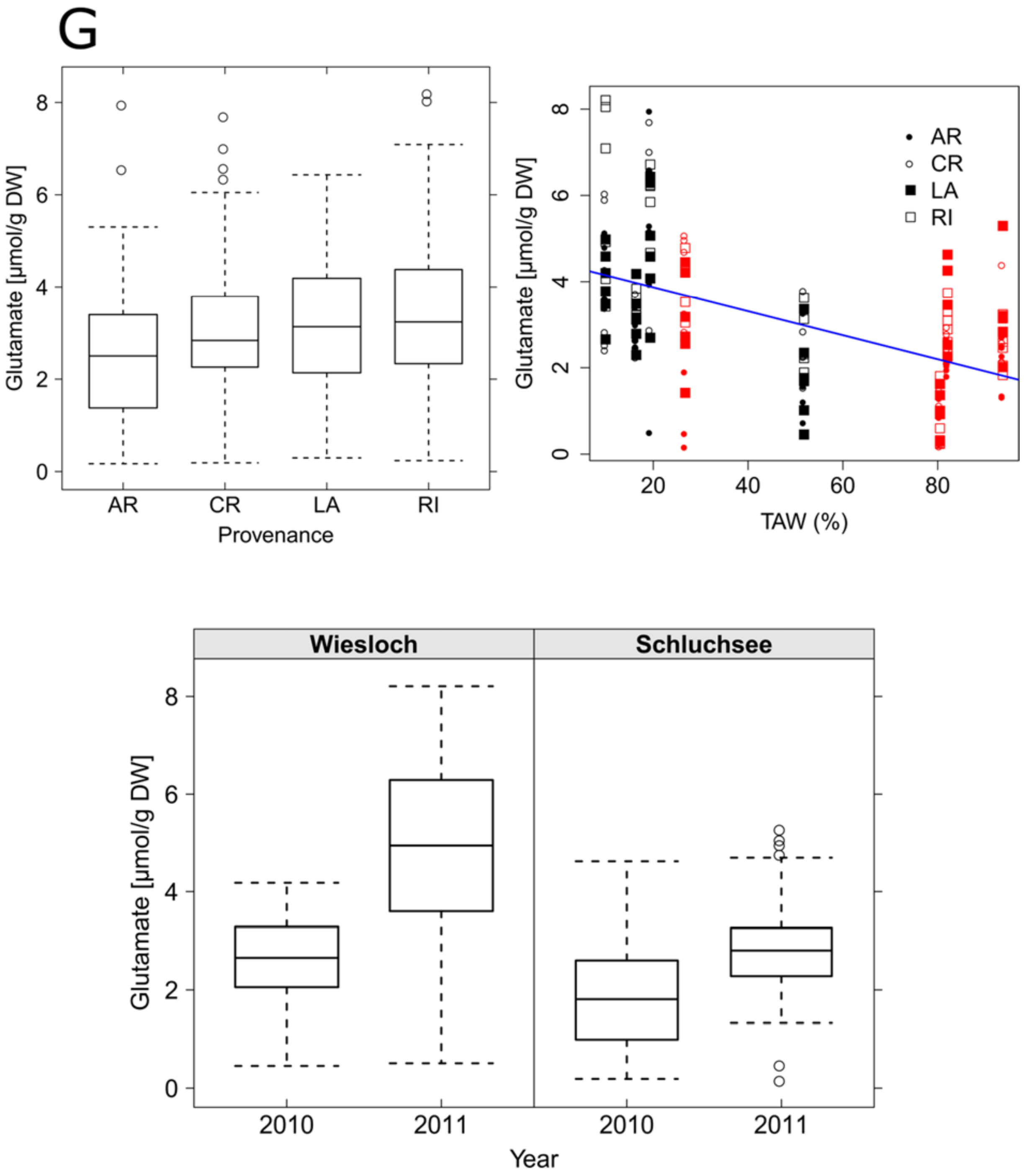
G
Glutamate [µmol/g DW]
AR
CR
LA
RI
Provenance
TAW (%)
Wiesloch
Schluchsee
2010
2011
Year

Figure S5 G
