# Supplementary material for: Foliar nitrogen metabolism of adult Douglas-fir trees is affected by soil water availability and varies little among provenances
Source: PLoS One. 2018 Mar 22;13(3):e0194684. doi: 10.1371/journal.pone.0194684 (PMC5864041; doi:10.1371/journal.pone.0194684)
Supplement: S6 Fig — (PDF) [file pone.0194684.s008.pdf]

Supporting Information

Foliar nitrogen metabolism of adult Douglas-fir trees is affected by soil water availability and varies little among provenances

Baoguo Du, Jürgen Kreuzwieser, Michael Dannenmann, Laura V. Junker, Anita Kleiber, Moritz Hess, Kirstin Jansen, Monika Eiblmeier, Arthur Gessler, Ulrich Kohnle, Ingo Ensminger, Heinz Rennenberg, Henning Wildhagen*

* Correspondence: Henning Wildhagen, HAWK University of Applied Sciences and Arts Hildesheim/Holzminden/Göttingen, Faculty of Resource Management, Büsgenweg 1A, 37077 Göttingen, Germany. Email: henning.wildhagen@hawk.de

S6 Figure

Correlation between needle total nitrogen (N) content and net photosynthetic assimilation rate (*A*) of adult Douglas-fir trees of four provenances (AR, Salmon Arm; CR, Conrad Creek; LA, Cameron Lake; RI, Santiam River) grown on field sites "Schluchsee" in south-western Germany. For a full presentation and discussion of data on *A* see Junker et al. (2017). Sampling was done in July 2010 and 2011. Per year, and provenance, *n*=3-5 trees were scored for both, total N content and *A*.

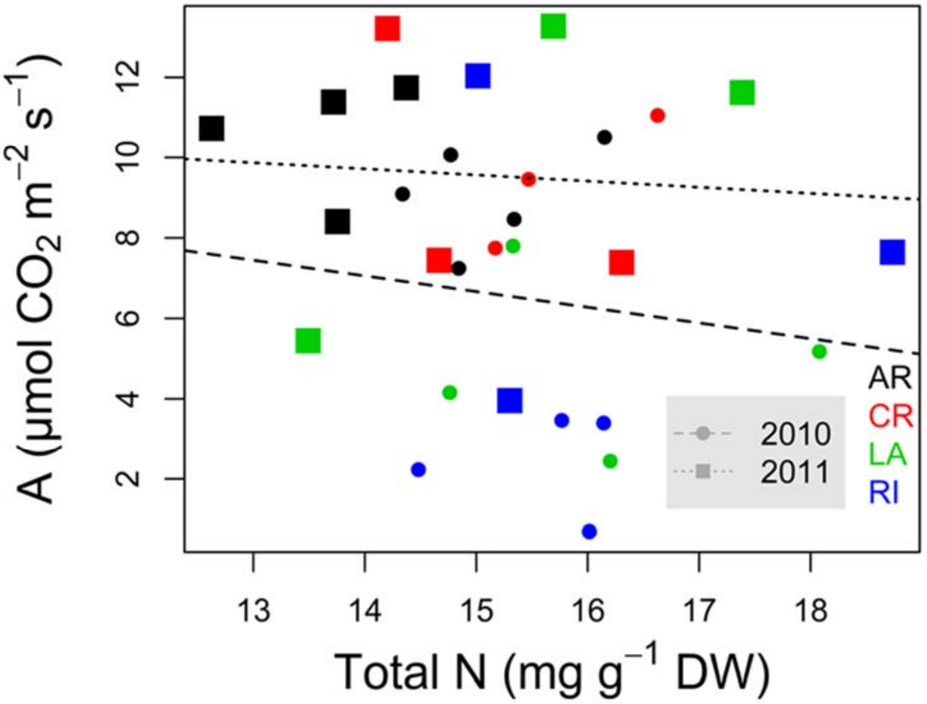

References

Junker LV, Kleiber A, Jansen K, Wildhagen H, Hess M, Kayler Z, Kammerer B, Schnitzler J-P, Kreuzwieser J, Gessler A (2017) Variation in short-term and long-term responses of photosynthesis and isoprenoid-mediated photoprotection to soil water availability in four Douglas-fir provenances. Sci Rep 7:40145.
