# Supplementary material for: Foliar nitrogen metabolism of adult Douglas-fir trees is affected by soil water availability and varies little among provenances
Source: PLoS One. 2018 Mar 22;13(3):e0194684. doi: 10.1371/journal.pone.0194684 (PMC5864041; doi:10.1371/journal.pone.0194684)

Supporting Information

Foliar nitrogen metabolism of adult Douglas-fir trees is affected by soil water availability and varies little among provenances

Baoguo Du, Jürgen Kreuzwieser, Michael Dannenmann, Laura V. Junker, Anita Kleiber, Moritz Hess, Kirstin Jansen, Monika Eiblmeier, Arthur Gessler, Ulrich Kohnle, Ingo Ensminger, Heinz Rennenberg, Henning Wildhagen*

* Correspondence: Henning Wildhagen, HAWK University of Applied Sciences and Arts Hildesheim/Holzminden/Göttingen, Faculty of Resource Management, Büsgenweg 1A, 37077 Göttingen, Germany. Email: henning.wildhagen@hawk.de

S7 Figure

Correlation between needle total nitrogen (N) content and tree height four of adult Douglas-fir trees of four provenances (AR, Salmon Arm; CR, Conrad Creek; LA, Cameron Lake; RI, Santiam River) grown on two field sites in south-western Germany. N contents determined in needles sampled in May and July of 2010 and 2011 were averaged for correlation analysis. Height of the trees was recorded in July 2011 following the needle sampling or when the growing season 2011 was finished. Colours of the symbols represent provenances: black, Salmon Arm; red, Conrad Creek; green, Cameron Lake; blue, Santiam River. Squared symbols: field site Schluchsee, circles: field site Wiesloch.

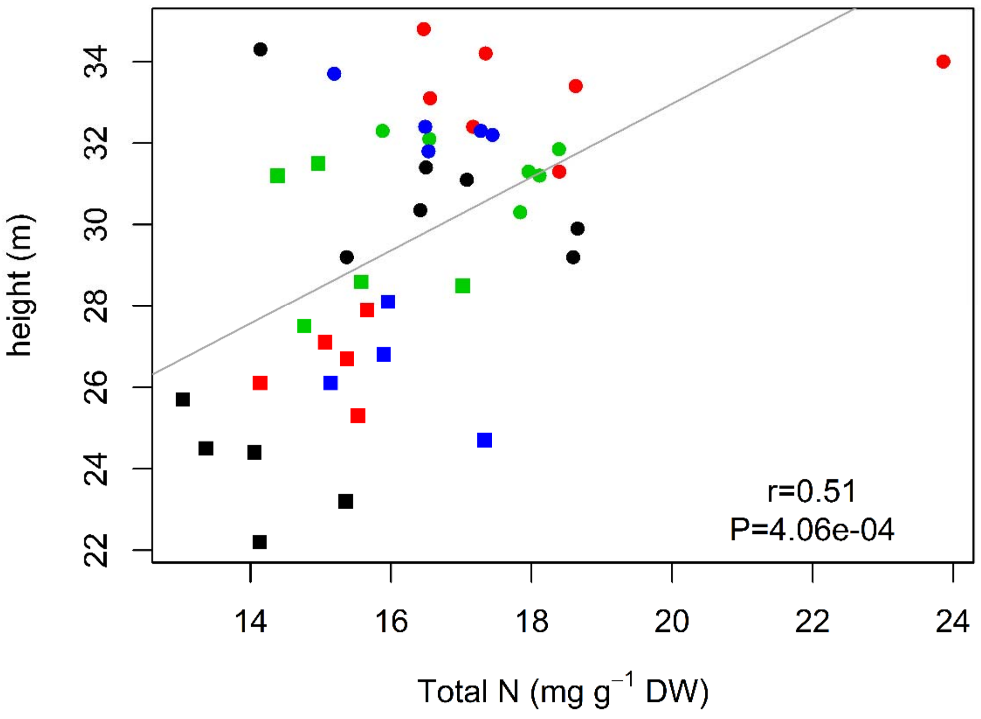

Supplement: S7 Fig — Trees were grown on two field sites in south-western Germany. (PDF) [file pone.0194684.s009.pdf]
